# Instantiating the Hash-then-evaluate paradigm: Strengthening PRFs, PCFs, and OPRFs

**DOI:** 10.1007/s12095-025-00825-3

**Published:** 2025-08-13

**Authors:** Chris Brzuska, Geoffroy Couteau, Christoph Egger, Pihla Karanko, Pierre Meyer

**Affiliations:** 1 Aalto University, Espoo, Finland; 2 Université Paris Cité, CNRS, IRIF, Paris, France; 3 Chalmers University of Technology and University of Gothenburg, Gothenburg, Sweden; 4 Aarhus University, Aarhus, Denmark

**Keywords:** Random oracle model, Extremely lossy functions, Pseudorandom functions, Pseudorandom correlation functions, 68P25, 94A60

## Abstract

We instantiate the hash-then-evaluate paradigm for pseudorandom functions (PRFs), $\textsf{PRF}(k,x):=\textsf{wPRF}(k,\textsf{RO}(x))$, which builds a PRF $\textsf{PRF}$ from a weak PRF $\textsf{wPRF}$ via a *public* pre-processing random oracle $\textsf{RO}$. In applications to secure multiparty computation (MPC), only the low-complexity $\textsf{wPRF}$ performs secret-depending operations. Our construction replaces $\textsf{RO}$ by $f(k_\textsf{H},\textsf{elf}(x))$, where *f* is a non-adaptive PRF and the key $k_\textsf{H}$ is *public* and thus known to the distinguishing adversary. We show that, perhaps surprisingly, several existing weak PRF candidates are plausibly also secure when their inputs are generated by $f(k_\textsf{H},\textsf{elf}(.))$. Firstly, analogous cryptanalysis applies (because pseudorandomness of *f* implies good statistical properties) and/or secondly an attack against the weak PRF with such pseudorandom inputs generated by *f* would imply surprising results such as key agreement from the hardness of the high-noise version of the Learning Parity with Noise (LPN) when implementing both $\textsf{wPRF}$ and *f* from this assumption. Our simple transformation of replacing $\textsf{RO}(\cdot )$ public pre-processing by $f(k_\textsf{H},\textsf{elf}(x))$ public pre-processing applies to the entire family of PRF-style functions. Specifically, we obtain results for oblivious PRFs, which are a core building block for password-based authenticated key exchange (PAKE) and private set intersection (PSI) protocols, and we also obtain results for pseudorandom correlation functions (PCF), which are a key tool for silent oblivious transfer (OT) extension.

## Introduction

The random oracle model (ROM) [16] is an idealised security model where all parties, honest or otherwise, are given oracle-access to the same uniformly chosen random function. Random oracles (ROs) model ideal hash functions and have found a plethora of applications in cryptography, including the Fiat-Shamir [25] transformation from 3-round interactive to non-interactive zero-knowledge proofs (NIZK), key-dependent message (KDM) security [17], adaptively secure garbled circuit [13], and many more. In this work, we are particularly interested in RO-based pre-processing of inputs as used, *e.g.* for password-based authenticated key exchange (PAKE) [21] and private set intersection (PSI) constructions [28]. Concretely, both PAKE and PSI first pre-process their inputs—a password for PAKE and database entries for PSI—by applying a RO and then use secure multi-party computation to evaluate a weak PRF on the RO result, a so-called *oblivious PRF* (OPRF) evaluation. This *hash-then-evaluate* paradigm, thus, pushes some of the complexity of the PRF $\textsf{PRF}(k,x):=\textsf{wPRF}(k,\textsf{RO}(x))$ into a purely offline phase, outside of the 2PC.

RO-based proofs for the hash-then-evaluate paradigm construct a reduction which emulates the random oracle and can therefore *observe* all queries to the RO as well as *program* the RO. In effect, the reduction chooses the mapping of the RO adaptively during the security experiment. Despite the practical use of the hash-then-evaluate paradigm, we do not know how to instantiate the RO in this transform.

### Extremely lossy functions

Zhandry [37] introduces the *non-black-box* framework of *extremely lossy functions (ELFs)* to build secure point function obfuscation with auxiliary input, polynomially-many hardcore bits for any one-way function and output intractable hash function—all inherently hard in the standard model—and later also deterministic encryption [38]. An ELF can be sampled either to be injective or lossy with a $\textsf{poly}$-size image, and yet, the injective mode and the lossy modes are (sufficiently) indistinguishable—if the adversary $\mathcal {A}$ is bounded by a *fixed* polynomial $\textsf{poly}^\prime (\lambda )$ as long as $\textsf{poly}^\prime (\lambda )\ll \textsf{poly}(\lambda )$ and $\tfrac{1}{\textsf{poly}(\lambda )}\ll \textsf{Adv}(\mathcal {A})$, where $\textsf{Adv}(\mathcal {A})$ denotes the adversary’s advantage. Since the notion is central to this paper (and so the introduction remains self-contained), we formally define extremely lossy functions already now.

### Definition 1

(Extremely Lossy Function ($\textsf{ELF}$), adapted from [37]) An *Extremely Lossy Function* ($\textsf{ELF}$) is a PPT algorithm $\textsf{ELF}.\textsf{Gen}$ which, on input a security parameter $1^\lambda$ and an image size $r\in [2^\lambda ]$, outputs a polynomial-time computable^1^ function $\textsf{elf} :\{0,1\}^\lambda \rightarrow \{0,1\}^*$ such that the following hold:

- **Injectivity.** $$\text {Pr}_{{\textsf{elf}{\leftarrow \!\!\!{\$}\,}}\,\textsf{ELF}.\textsf{Gen}(1^\lambda ,2^\lambda )}{\left[ |\textsf{elf}(\{0,1\}^\lambda )|=2^\lambda \right] }=1-{\mathsf {negl(\lambda )}}$$.
- **Lossiness.** $$\forall c_\textsf{elf}\in \mathbb {N}$$: $$\text {Pr}_{\textsf{elf}{\leftarrow \!\!\!{\$}\,}\,\textsf{ELF}.\textsf{Gen}(1^\lambda ,\lambda ^{c})}{\left[ |\textsf{elf}(\{0,1\}^\lambda )|\le \lambda ^{c}\right] }=1-\mathsf {negl(\lambda )}$$.
- **Indistinguishability.** $\forall a,t\in \mathbb {N}, \exists c\in \mathbb {N}$ s.t. for all $\mathcal {A}$ running in time $\le \lambda ^{t}$:
  $$ \left| \text {Pr}_{ \textsf{elf}{\leftarrow }{{\$}}\textsf{ELF}.\textsf{Gen}(1^{\lambda ,}2^{\lambda })}\left[ {1 \!=\! \mathcal {A}(1^\lambda ,\textsf{elf})}\right] \!-\! \text {Pr}_{\textsf{elf}{\leftarrow }{{\$}}\textsf{ELF}.\textsf{Gen}(1^{\lambda },\lambda ^{c})}\left[ {1 = \mathcal {A}(1^\lambda ,\textsf{elf})}\right] \right|< \lambda ^{-a}. $$
- ***Enumerable image.*** $\forall c_\textsf{elf}\in \mathbb {N}$, $\exists$ PPT$(\lambda ^{c}) \, \mathcal {C}:$
  $$\begin{aligned} \text {Pr}_{\textsf{elf}{\leftarrow }{{\$}}\textsf{ELF}.\textsf{Gen}(1^{\lambda },\lambda ^{c})} \left[ {\textsf{elf}(\{0,1\}^{\lambda })\subseteq \mathcal {C}(1^\lambda ,\textsf{elf})}\right] \ge 1-\mathsf {negl(\lambda )}~. \end{aligned}$$

The *non-black-box property* (via dependency on the adversary’s runtime) as well as the polynomial-time enumerability of the image space of an ELF in lossy mode are two powerful tools for instantiating random oracles.

### Contributions

Our main contribution is to instantiate the hash-and-evaluate paradigm for a wide range of *PRF-like* objects. We start by instantiating this approach for PRFs, which has direct implications for low-complexity OPRFs. Our techniques also apply to *pseudorandom correlation functions* (PCFs) [5], which allow parties to locally expand short correlated keys into large amounts of correlated randomness which can then be used to perform secure multiparty computation efficiently.

Our instantiation of hash-then-evaluate $\textsf{sPRF}(k, \cdot ) = \textsf{wPRF}(k, \textsf{RO}(\cdot ))$ replaces the random oracle by the hash-function $\textsf{H}(.):=f(k_\textsf{H},\textsf{elf}(.))$ where *f* is a (non-adaptive^2^) PRF and the key $k_\textsf{H}$ is *public*. That is, we replace the $\textsf{RO}$ by a *public* pre-processing phase which does not depend on the secret key *k* and hence, in secure multi-party computation applications, does not need to be securely evaluated, but can be performed locally.

One caveat is that the outputs of $\textsf{H}(.)$ are not random and $\textsf{wPRF}$ expects random inputs. However, when $k_\textsf{H}$ is not given, they are at least *pseudorandom*. Thus, we strengthen the security requirements on the weak PRF in the spirit of Pietrzak and Sjödin [35] who strengthen weak PRFs to secret-coin weak PRFs where the adversary is a sampler-distinguisher, *i.e.* the adversary first samples inputs (non-adaptively), conditioned on them being uniformly random, and then tries to distinguish PRF outputs from random. This is stronger than a weak PRF, because, e.g., the adversary can sample a uniformly random group element *h* by first sampling a uniformly random exponent *x* and then returning $h=g^x$. We strengthen [35]’s definition of a secret-coin weak PRF into a pseudorandom-input PRF (PI-PRF) which is secure as long as the sampler-distinguisher chooses *pseudorandom* values. Specifically, we are interested in (non-adaptive) samplers which first sample *t* (arbitrarily distributed, but distinct) values $x_1,..,x_t$, then sample a key $k_{\textsf{H}}$ and return $z_1=f(k_{\textsf{H}},x_1),..,z_t=f(k_{\textsf{H}},x_t)$, where *f* is a (strong) PRF. If $\textsf{wPRF}(k,z_i)$ is secure for a secret uniformly random key $k_{\textsf{H}}$ and inputs $z_i$ from a distribution of the aforementioned shape, then we call $\textsf{wPRF}$ a $\textsf{PI}_{f}-\textsf{PRF}$ (cf. Definition 12).

To prove strong PRF security, we additionally pre-process the inputs by an ELF. Our core observation is that the set of image values $\textsf{Im}(\textsf{elf})$ in lossy mode is efficiently enumerable and independent of $k_\textsf{H}$. Hence, evaluating a PRF *f* with public-key $k_\textsf{H}$ on $\textsf{Im}(\textsf{elf})$ yields a set of suitable inputs for the $\mathsf {PI\text {-}PRF}$.

#### The need for a CRS

We replace $$\textsf{sPRF}(k,x):= \textsf{wPRF}(k,\textsf{RO}(x))$$ by $$\textsf{sPRF}(k,x):= \mathsf {PI\text {-}PRF}(k,$$$$\textsf{H}(k_\textsf{H},x))$$, where $$k_\textsf{H}$$ is a uniformly random public value. Thus, we need to incorporate $$k_\textsf{H}$$ into our syntax and security definition of a strong PRF such that $$k_\textsf{H}$$ is given to the adversary. Alternatively, we could have $$k_\textsf{sPRF}^\prime :=(k_\textsf{sPRF},k_\textsf{H})$$, but including $$k_\textsf{H}$$ into the secret-key means, we would prove too weak a security notion, where $$\mathcal {A}$$ does not see the key; this security notion would not suffice to establish that *public* preprocessing by $$\textsf{H}(k_\textsf{H},x)$$ is secure, as required by our applications (distributed PRFs, oblivious PRFs and PCFs). Hence, we include the public value $$k_\textsf{H}$$ as additional variable into our syntax and model explicitly and call $$k_\textsf{H}$$ a *common random string (CRS)* in accordance with [10]. We refer to a PRF which takes a key, and input and an additional *public* value $$\textsf{crs}$$ a *PRF in the CRS model*. CRS model is not a strong limitation for the aforementioned applications that anyway have a public pre-processing phase, especially when the alternative is the RO model, which essentially already assumes the existence of globally accessible setup.

#### Contribution 1

(Instantiating hash-then-evaluate) We show that the following is a strong PRF in the Common Reference String model (the CRS contains $k_\textsf{H}$ and $\textsf{elf}$):

$$\textsf{sPRF}(k,x):= \mathsf {PI\text {-}PRF}(k,\textsf{naPRF}(k_\textsf{H},\textsf{elf}(x)))~.$$

We also show analogous result for oblivious PRFs and for PCFs (without the need for a CRS 3).

#### Cryptanalysis and win-win results

Our second main result is the observation that the cryptanalysis of several weak PRFs [5, 7, 14] actually also applies when the inputs are pseudorandom, namely, for as long as they satisfy suitable statistical properties—or at least, that violating the security of these wPRFs for *pseudorandom* inputs would have interesting consequences. In slightly more detail, we consider the weak PRF of [14], which is at the heart of one of the most efficient (to date) oblivious PRF protocols [23], and the weak PRFs of [5, 7], which are at the heart of the most efficient PCFs known to date. For each of these candidates, we analyze the most natural families of attacks, and obtain the following results:

#### Contribution 2

(Cryptanalysis and Surprising Implications) We put forward evidence of existing wPRF candidates being plausibly PI-PRF candidates.

- **Statistical query algorithms.** [14] show that when inputs are chosen uniformly randomly, their candidates resists all *statistical query attacks*, one of the most common types of attacks against low-complexity PRF candidates. We strengthen their analysis to the case of pseudorandom inputs.
- **Large pseudodistance.** It is an open question whether codes with low (sublinear) minimum distance can be computationally indistinguishable from codes which have large (linear) distance. The existence of such codes with large pseudodistance would have interesting consequences for low-complexity cryptographic hash functions [3]. We show that either there exists an efficiently sampleable family of linear codes with large pseudo-distance, or the wPRF candidates of [5, 7] are secure against attacks from the *linear test framework* (the main framework used to study the security of wPRFs from LPN-style assumptions, which both these candidates are) even when the wPRF inputs are pseudorandom.
- **Key Agreement from high-noise LPN.** As a final plausibility check, we show that for a $\textsf{wPRF}$ from high-noise LPN, there must exists a PRF *f* such that $\textsf{wPRF}$ is also a $\textsf{PI}_f\text {-}\textsf{wPRF}$ or else, we would obtain the very surprising result of (infinitely often correct) key agreement from high-noise LPN.

#### Organisation

We instantiate the hash-then-evaluate paradigm for PRFs in Section 3.3, for OPRFs in Section 4.1, and finally for PCFs in Section 4.2. Finally, we present our cryptanalysis and reflection on the plausibility of existing wPRF/wPCFs being PI-PRFs/PI-PCFs in Section 5.

## Preliminaries

### Probabilities

Given $p \in (0,1)$, we let $\textsf{Ber}_p$ denote the Bernoulli distribution with parameter *p* (*i.e.*, $\textsf{Ber}_p$ outputs 1 with probability *p* and 0 with probability $1-p$). We recall the definition of the bias of a distribution:

#### Definition 2

(Bias of a Distribution) Given a distribution $\mathcal {D}$ over $\mathbb {F}{}^n$ and a vector $\textbf{u} \in \mathbb {F}{}^n$, the bias of $\mathcal {D}$ with respect to $\textbf{u}$, denoted $\textsf{bias}_{\textbf{u}}(\mathcal {D})$, is equal to

$$\begin{aligned} \textsf{bias}_{\textbf{u}}(\mathcal {D}) = \left| \Pr _{\textbf{x} \sim \mathcal {D}}[\textbf{u} \cdot \textbf{x}^\intercal =0] - \Pr _{\textbf{x} \sim \mathcal {U}_n}[\textbf{u}\cdot \textbf{x}^\intercal =0] \right| = \left| \Pr _{\textbf{x} \sim \mathcal {D}}[\textbf{u} \cdot \textbf{x}^\intercal =0] - \frac{1}{|\mathbb {F} |} \right| , \end{aligned}$$

where $\mathcal {U}_n$ denotes the uniform distribution over $\mathbb {F}{}^n$. The bias of $\mathcal {D}$, denoted $\textsf{bias}(\mathcal {D})$, is the maximum bias of $\mathcal {D}$ with respect to any nonzero vector $\textbf{u}$.

The piling-up lemma provides a standard way to compute the bias of a distribution constructed as the XOR of i.i.d. samples:

#### Lemma 1

(Piling-up lemma) For $0< \mu < \frac{1}{2}$ and random variables $(X_1, \dots , X_t)$ i.i.d. to $\textsf{Ber}_\mu$, denoting $X = \bigoplus _{i=1}^t X_i$, it holds that

$$ \Pr \left[ X = 0\right] = \frac{1}{2} \cdot \left( 1 + (1 - 2\mu )^t\right) .$$

In other terms, denoting $\mathcal {D}_X$ the distribution of *X*, we have

$$ \textsf{bias}(\mathcal {D}_X) = \frac{(1-2\mu )^t}{2}. $$

Eventually, we will need the following standard concentration inequality:

#### Lemma 2

Bienaymê-Chebyshev Inequality Let *X* be a random variable with finite expected value $\mu$ and finite nonzero variance $\sigma ^2$. Then for any $k > 0$,

$$\begin{aligned} \Pr [|X - \mu | \ge k\sigma ] \le \frac{1}{k^2}. \end{aligned}$$

### Pseudorandom correlation functions

At a high level, a *pseudorandom correlation function* (PCF) cryptographically compresses (superpolynomial-size) correlated random strings from some ideal correlation, *e.g.* generating long vectors of Beaver triples [9]^4^, down to short keys. Given a key, it should be possible to incrementally recover parts of the long string, *e.g.* evaluating the PCF key at position *i* should yield a party’s share of the $i^\text {th}$ Beaver triple. Prior works have considered three different flavours of PCFs, from weakest to strongest: *weak* PCFs (wPCF), *non-adaptive* PCFs (naPCF), and *strong* PCFs (sPCF). Intuitively, and analogously to their PRF counterparts, security is guaranteed (*e.g.* the pseudorandom Beaver triples are “safe to use”) when evaluating the PCF keys at random (resp. non-adaptively chosen, resp. any) points. Note that contrary to PRFs, the PCF literature treats *weak* PCFs (security w.r.t. random inputs) as the default PCF which is motivated in part by [5, Theorem 4.5], which shows that the hash-then-evaluate paradigm can be used to turn a weak PCF into a strong one.

For technical reasons, and in order to provide a meaningful definition of PCF for infinite families of finite correlations, we only consider *reverse sampleable* correlations (Definition 3). We refer to [5, Section 4] for more details.

#### Definition 3

(Reverse-Sampleable Correlation, [4]) Let $1\le \ell _0(\lambda ),\ell _1(\lambda )\le \textsf{poly}(\lambda )$ be output-length functions. Let $\mathcal {Y}$ be a probabilistic algorithm on input $1^\lambda$, returns a pair of outputs $(y_0,y_1)\in \{0,1\}^{\ell _0(\lambda )}\times \{0,1\}^{\ell _1(\lambda )}$, defining a correlation on the outputs.

We say that $\mathcal {Y}$ defines a *reverse-sampleable* correlation if there exists a PPT algorithm $\textsf{RSample}$ which takes as input $1^\lambda$, $\sigma \in \{0,1\}$, and $y_\sigma \in \{0,1\}^{\ell _\sigma (\lambda )}$, and outputs $y_{1-\lambda }^{\ell _{1-\sigma }(\lambda )}$, such that for all $\sigma \in \{0,1\}$

$$\begin{aligned} \begin{aligned}&\{(y_0,y_1):(y_0,y_1)\,{\leftarrow \!\!\!{\$}\,}\,\mathcal {Y}(1^\lambda )\}\text { and }\\&\qquad \qquad \qquad \qquad \{(y_0,y_1):(y_0',y_1')\,{\leftarrow \!\!\!{\$}\,}\,\mathcal {Y}(1^\lambda ),y_\sigma \leftarrow y'_\sigma ,y_{1-\sigma }\leftarrow \textsf{RSample}(1^\lambda ,\sigma ,y_\sigma )\}~. \end{aligned} \end{aligned}$$

are statistically close.

All the different flavours of PCF admit the same syntax, which we describe in Definition 4.

#### Definition 4

(Pseudorandom Correlation Function – Syntax [5, Definition 4.3]) Let $\mathcal {Y}$ be a reverse-sampleable correlation with output length functions $\ell _0(\lambda ),\ell _1(\lambda )$ and let $\lambda \le n(\lambda )\le \textsf{poly}(\lambda )$ be an input length function. Syntactically, a *pseudorandom correlation function* is a pair of algorithms $\textsf{PCF}=(\textsf{PCF}.\textsf{Gen},\textsf{PCF}.\textsf{Eval})$ with the following syntax:

- $\textsf{wPCF}.\textsf{Gen}(1^\lambda )$ is a probabilistic polynomial time algorithm that on input $1^\lambda$, outputs a pair of keys $(k_0,k_1)$; we assume that $\lambda$ can be inferred from the keys.
- $\textsf{wPCF}.\textsf{Eval}(\sigma ,k_\sigma ,x)$ is a deterministic polynomial time algorithm that on input $\sigma \in \{0,1\}$, key $k_\sigma$ and input value $x\in \{0,1\}^{n(\lambda )}$, outputs a value $y_\sigma \in \{0,1\}^{\ell _\sigma (\lambda )}$.

### (Weak) Pseudorandom Correlation Function (wPCF)

A PCF (with the syntax of Definition 4) is said to be a secure *weak pseudorandom correlation function (wPCF)* if it satisfies the properties of Definitions 5 and 6. At a high level, the property of *(weak) pseudorandom* $\mathcal {Y}$*-correlated outputs* states that the evaluations of the PCF (on truly random points) should look like samples from the ideal distribution $\mathcal {Y}$ *from the point of view of an external adversary (who does not hold a PCF key)*. The *(weak) PCF security* property captures that a player holding a PCF key and seeing the other PCF key’s evaluation at random points should learn “nothing about the other PCF key, except for its evaluation at those points”.

#### Definition 5

**Fig. 1:**
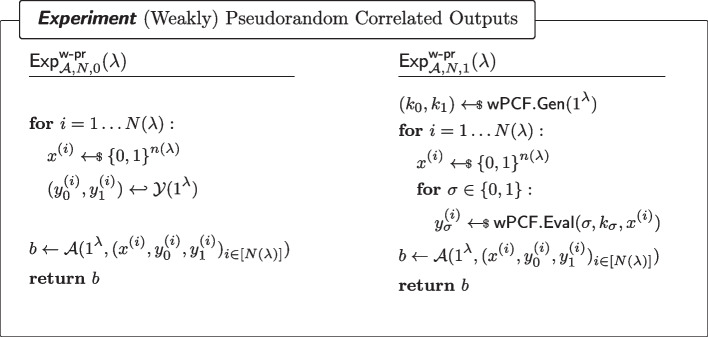
(Weakly) Pseudorandom $\mathcal {Y}$-correlated outputs of a wPCF

((Weakly) pseudorandom $\mathcal {Y}$-correlated outputs of a PCF) For every non-uniform adversary $\mathcal {A}$ of size $B(\lambda )$, it holds that for all sufficiently large $\lambda$,

$$\begin{aligned} \left| \Pr [\textsf{Exp}^\mathsf {w{\text{- }}pr}_{\mathcal {A},N,0}(\lambda )=1]-\Pr [\textsf{Exp}^\mathsf {w{\text{- }}pr}_{\mathcal {A},N,1}(\lambda )=1]\right| \le \mathsf {negl(\lambda )}\end{aligned}$$

where $\textsf{Exp}^\mathsf {w{\text{- }}pr}_{\mathcal {A},N,b}$ ($b\in \{0,1\}$) is defined as in Fig. 1. In particular, the adversary is given access to $N(\lambda )$ samples.

#### Definition 6

**Fig. 2:**
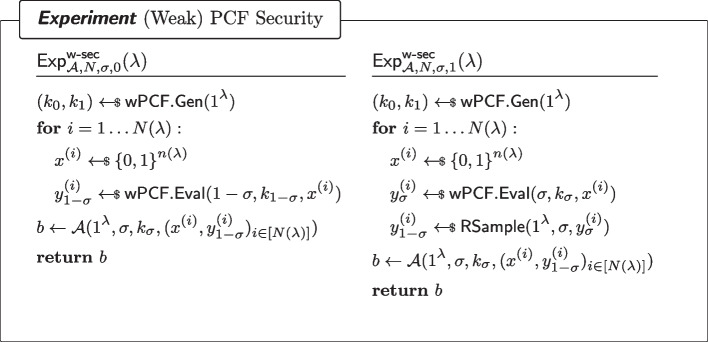
Security of a wPCF. $\textsf{RSample}$ reverse-samples $\mathcal {Y}$ as in Definition 3

((Weak) PCF Security) For every $\sigma \in \{0,1\}$ and every non-uniform adversary $\mathcal {A}$ of size $B(\lambda )$, it holds that for all sufficiently large $\lambda$,

$$\begin{aligned} |\Pr [\textsf{Exp}^\mathsf {w{\text{- }}sec}_{\mathcal {A},N,\sigma ,0}(\lambda )=1]-\Pr [\textsf{Exp}^\mathsf {w{\text{- }}sec}_{\mathcal {A},N,\sigma ,1}(\lambda )=1]|\le \mathsf {negl(\lambda )}\end{aligned}$$

where $\textsf{Exp}^\mathsf {w{\text{- }}sec}_{\mathcal {A},N,\sigma ,b}$ ($b\in \{0,1\}$) is defined as in Fig. 2. In particular, the adversary is given access to $N(\lambda )$ samples (or simply *N* if there is no ambiguity).

### Non-Adaptive Pseudorandom Corr. Function (naPCF).

A PCF $\textsf{naPCF}=(\textsf{naPCF}.\textsf{Gen},\textsf{naPCF}.\textsf{Eval})$ (with the syntax of Definition 4) is a secure *non-adaptive pseudorandom correlation function (naPCF)* if it satisfies the properties of Definitions 7 and 8. These properties are analogous to the weak counterpart, but hold on non-adaptively chosen queries, instead of only on truly random ones.

#### Definition 7

**Fig. 3:**
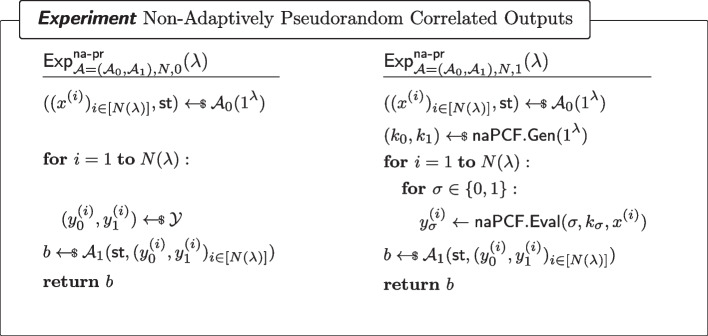
Non-Adaptively Pseudorandom $\mathcal {Y}$-correlated outputs of a naPCF

(Non-adaptively pseudorandom $\mathcal {Y}$-correlated outputs) For non-uniform adversary $\mathcal {A}=(\mathcal {A}_0,\mathcal {A}_1)$ of size $B(\lambda )$ asking at most $N(\lambda )$ non-adaptive queries to the oracle $\mathcal {O}_b(\cdot )$ (as defined in Fig. 3) and all sufficiently large $\lambda$,

$$\begin{aligned} \left| \Pr [\textsf{Exp}^\mathsf {na{\text{- }}pr}_{\mathcal {A}=(\mathcal {A}_0,\mathcal {A}_1),N,0}(\lambda )=1]-\Pr [\textsf{Exp}^\mathsf {na{\text{- }}pr}_{\mathcal {A}=(\mathcal {A}_0,\mathcal {A}_1),N,1}(\lambda )=1]\right| \le \mathsf {negl(\lambda )}\end{aligned}$$

where $\textsf{Exp}^\mathsf {na{\text{- }}pr}_{\mathcal {A}=(\mathcal {A}_0,\mathcal {A}_1),N,b}$ ($b\in \{0,1\}$) is defined as in Fig. 3.

**Fig. 4:**
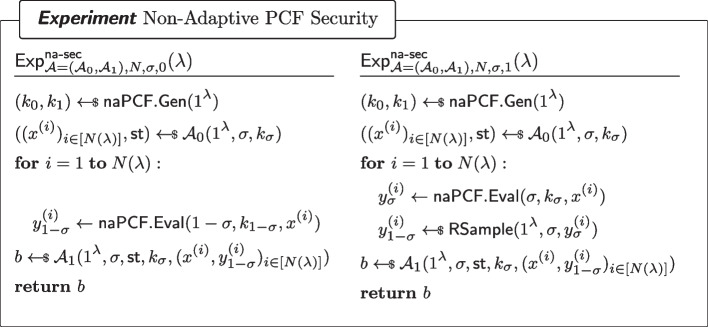
Security of a non-adaptive PCF. Here, $\textsf{RSample}$ is the algorithm for reverse sampling $\mathcal {Y}$ as in Definition 3

#### Definition 8

(Non-Adaptive PCF Security) For every $\sigma \in \{0,1\}$, every non-uniform adversary $\mathcal {A}=(\mathcal {A}_0,\mathcal {A}_1)$ of size $B(\lambda )$ and for all sufficiently large $\lambda$,

$$\begin{aligned} \left| \Pr [\textsf{Exp}^\mathsf {na{\text{- }}sec}_{\mathcal {A}=(\mathcal {A}_0,\mathcal {A}_1),N,\sigma ,0}(\lambda )=1]-\Pr [\textsf{Exp}^\mathsf {na{\text{- }}sec}_{\mathcal {A}=(\mathcal {A}_0,\mathcal {A}_1),N,\sigma ,1}(\lambda )=1]\right| \le \mathsf {negl(\lambda )}\end{aligned}$$

where $\textsf{Exp}^\mathsf {na{\text{- }}sec}_{\mathcal {A}=(\mathcal {A}_0,\mathcal {A}_1),N,\sigma ,b}$ ($b\in \{0,1\}$) is defined as in Fig. 4.

**Fig. 5:**
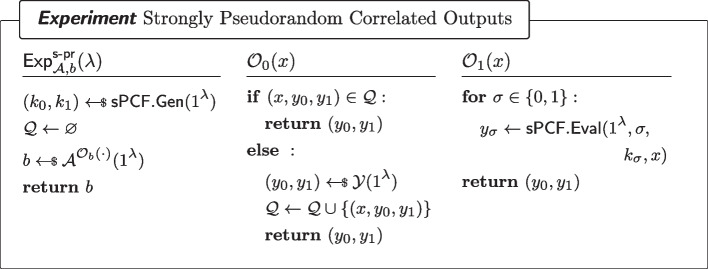
Strongly Pseudorandom $\mathcal {Y}$-correlated outputs of a sPCF

**Fig. 6:**
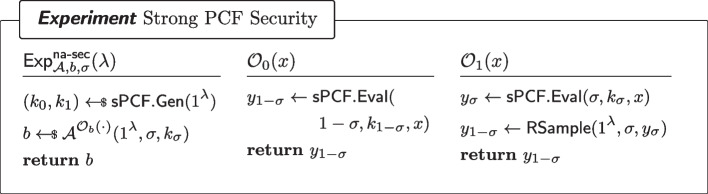
Security of a strong PCF. Here, $\textsf{RSample}$ is the algorithm for reverse sampling $\mathcal {Y}$ as in Definition 3

### Strong Pseudorandom Correlation Function (sPCF)

A PCF $\textsf{sPCF}=(\textsf{sPCF}.\textsf{Gen},\textsf{sPCF}.\textsf{Eval})$ (with the syntax of Definition 4) is said to be a secure *strong pseudorandom correlation function (sPCF)* if it satisfies the properties of Definitions 9 and 10. These properties are analogous to the non-adaptive counterpart, but hold on adaptively chosen queries, instead of only on non-adaptive ones.

#### Definition 9

(Strongly pseudorandom $\mathcal {Y}$-correlated outputs) For every non-uniform adversary $\mathcal {A}$ of size $B(\lambda )$ asking at most $N(\lambda )$ queries to the oracle $\mathcal {O}_b(\cdot )$ (as defined in Fig. 5), it holds that for all sufficiently large $\lambda$,

$$\begin{aligned} \left| \Pr [\textsf{Exp}^\mathsf {s{\text{- }}pr}_{\mathcal {A},0}(\lambda )=1]-\Pr [\textsf{Exp}^\mathsf {s{\text{- }}pr}_{\mathcal {A},1}(\lambda )=1]\right| \le \mathsf {negl(\lambda )}\end{aligned}$$

where $\textsf{Exp}^\mathsf {s{\text{- }}pr}_{\mathcal {A},b}$ ($b\in \{0,1\}$) is defined as in Fig. 5.

#### Definition 10

(Strong PCF Security) For every $\sigma \in \{0,1\}$ and every non-uniform adversary $\mathcal {A}$ of size $B(\lambda )$ asking at most $N(\lambda )$ queries to the oracle $\mathcal {O}_b(\cdot )$ (as defined in Fig. 6), it holds that for all sufficiently large $\lambda$,

$$\begin{aligned} |\Pr [\textsf{Exp}^\mathsf {s{\text{- }}sec}_{\mathcal {A},0,\sigma }(\lambda )=1]-\Pr [\textsf{Exp}^\mathsf {s{\text{- }}sec}_{\mathcal {A},1,\sigma }(\lambda )=1]|\le \mathsf {negl(\lambda )}\end{aligned}$$

where $\textsf{Exp}^\mathsf {s{\text{- }}sec}_{\mathcal {A},\sigma }$ is defined as in Fig. 6.

## Instantiating Hash-then-Evaluate PRFs

The *Hash-then-Evaluate* paradigm relies on the fact that, if $\textsf{wPRF}$ is a weak PRF and $\textsf{RO}$ is a *programmable* random oracle, then $\textsf{wPRF}\circ \textsf{RO}$ is a strong PRF. This transformation truly shines in distributed settings (*e.g.* distributed PRFs, oblivious PRFs, and correlated PRFs—better known as pseudorandom correlation functions (PCF)—), where the hash function is applied locally in an *input pre-processing phase*, thereby limiting the use of expensive compilers (such as *secure multiparty computation*) to securely evaluate only *weak* PRFs, which admit significantly lower-complexity candidates than strong PRFs.

### Programmability

The random oracle proof of the transformation $\textsf{PRF}:=\textsf{wPRF}\circ \textsf{RO}$ relies on programming because, in the weak PRF game, evaluation points $z_1,..,z_t$ are sampled uniformly at random by the experiment. In turn, in the strong PRF security game, the adversary gets to choose the inputs $x_1,..,x_t$ and therefore, the reduction needs to program the $\textsf{RO}$ such that $\textsf{RO}(x_i)=z_i$. We circumvent the need for programmeability by (1) strengthening the $\textsf{wPRF}$ to a $\mathsf {PI_f{\text{- }}PRF}$ which is secure under pseudorandom inputs generated by $f(k_{\textsf{H}},\cdot )$, where the inputs to $f(k_{\textsf{H}},\cdot )$ are chosen independently of $k_{\textsf{H}}$ and then (2) generate the inputs to $f(k_{\textsf{H}},\cdot )$ by querying the entire image $\textsf{Im}(\textsf{elf})$ in the reduction to $\mathsf {PI_f{\text{- }}PRF}$ security, which we can do in the security proof only after having made $\textsf{elf}$ extremely lossy so that image $\textsf{Im}(\textsf{elf})$ is polynomial-size. No matter which value $x_i$ the adversary later chooses (adaptively) in the (strong) PRF game, the value of $\textsf{wPRF}(k,\cdot )$ on $f(k_{\textsf{H}},\textsf{elf}(x_i))$ will be known to the reduction, because $\textsf{elf}(x_i)\in \textsf{Im}(\textsf{elf})$. Therefore the programming is not needed, because the correct values are known beforehand.

### Construction

We describe our construction in a modular way. Pre-processing only by a non-adaptive PRF boosts security of a pseudorandom-input PRF to a non-adaptive PRF in the CRS model. Additionally pre-processing the input by an ELF boosts security from non-adaptive (in the CRS model) to strong PRF security (in the CRS model). While the 2nd step is, so far, of purely theoretical nature, the first step is very lightweight (both in terms of efficiency and assumptions) and in fact practically deployable. Concretely, it can be used whenever the (non-adaptive) PRF is evaluated on points *according to a pre-agreed upon order*. In particular, this is how non-adaptive PCFs are used, and thus, non-adaptive security suffices for MPC applications.

### Pseudorandom-Input PRF (PI-PRF)

We now formalise *pseudorandom-input PRFs* $\mathsf {PI-PRF}$ and $\mathsf {PI_f{\text{- }}PRF}$, which produce pseudorandom values as long as inputs are sampled by an admissible sampler ($\Rightarrow$ pseudorandom inputs) and an *f*-admissible sampler ($\Rightarrow$ pseudorandom inputs by applying *f* to non-adaptively sampled values), respectively.

#### Definition 11

(Admissible Sampler) Let $\textsf{len}$ and *N* be polynomials in $\lambda$. A polynomial-time sampler $\textsf{Sam}_{\lambda ,N}:\{0,1\}^\textsf{len} \rightarrow \{0,1\}^{N \times \lambda }$ is admissible, if for all probabilistic polynomial-time (PPT) $\mathcal {A}$

$$\begin{aligned} |\text {Pr}_{r\,\leftarrow \!\!\$\,\{0,1\}^{\textsf{len}}}{1=\mathcal {A}(\textsf{Sam}_{\lambda ,N}(r))} - \text {Pr}_{\forall i: x_i \,\leftarrow \!\!\$\, \{0,1\}^\lambda }{1=\mathcal {A}(x_1,...,x_N)}| = {negl}(\lambda ), \end{aligned}$$

where $r {\leftarrow }{{\$}}\{0,1\}^{\textsf{len}}$ denotes uniform sampling from $\{0,1\}^{\textsf{len}}$. We say that $\textsf{Sam}_{\lambda ,N}$ is *f*-admissible if there exists a polynomial-time sampler $\textsf{Sam}^{\textsf{na}}_{\lambda ,N}:\{0,1\}^* \rightarrow \{0,1\}^{N \times \lambda }$ such that $\textsf{Sam}_{\lambda ,N}(r=(r^\prime ||k)):=f(k,\textsf{Sam}^{\textsf{na}}_{\lambda ,N}(r^\prime ))$. Sometimes we might not write the randomness *r* explicitly, but instead consider $\textsf{Sam}_{\lambda ,N}$ as PPT adversary that samples *r* uniformly itself and does not take any input. We write $\textsf{R}:=\textsf{R}(\textsf{Sam}_{\lambda ,N}):= |r|$ for the length of the randomness.

In the definition of admissible sampler, the adversary against $\textsf{Sam}$ does not see the sampler’s randomness *r* (only $\textsf{Sam}$’s output). In turn, in the following definition of PI-PRF, the adversary against PI-PRF receives *r*. We now define weak PRFs, PI-PRFs, non-adaptive PRFs and strong PRFs. For the latter two, we also define a variant in the CRS model, as previously described. The non-adaptive sampler in the CRS model does not get to see the CRS, but for applications where the evaluation points are pre-agreed anyway, this security level suffices.

#### Definition 12

(Pseudorandom Functions (PRF)) A pseudorandom function is a polynomial-time computable collection of functions $(f_\lambda )_{\lambda \in \mathbb {N}}$ with

$$\begin{aligned} f_\lambda :\{0,1\}^\lambda \times \{0,1\}^\lambda&\rightarrow \{0,1\}^\lambda \\ \text {or}\\ f_\lambda :\{0,1\}^\lambda \times \{0,1\}^{\textsf{C}(\lambda )}\times \{0,1\}^\lambda&\rightarrow \{0,1\}^\lambda \text { when} f \text {is in the CRS model.} \end{aligned}$$

We usually omit the index $\lambda$. We say that *f* is a

**Fig. 7:**
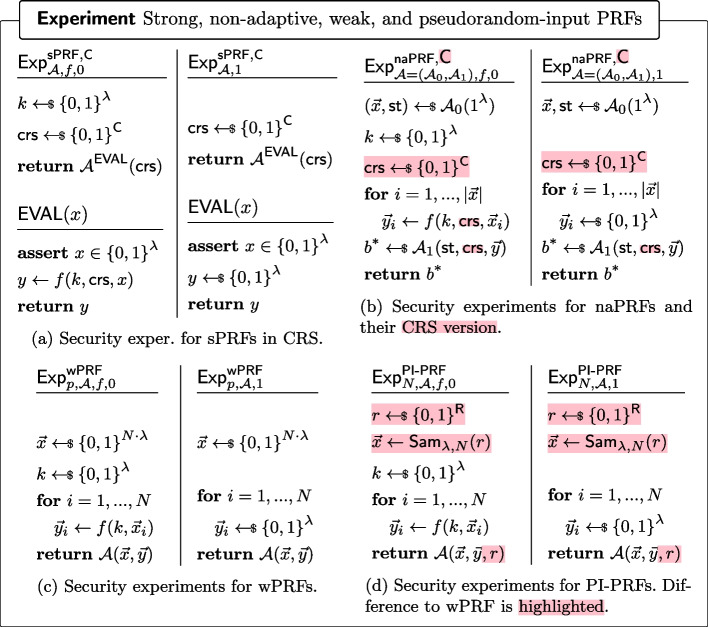
Security experiments of strong, non-adaptive, weak, and pseudorandom-input PRFs. $\mathcal {A}^{\text{\O }{Eval}}$ denotes that adversary $\mathcal {A}$ can adaptively query the $\text{\O }{Eval}$ oracle

- **strong PRF** [26] **in the CRS model** if for all PPT $\mathcal {A}^{\textsf{EVAL}}$ who never queries the same input *x* twice to the oracle ${\textsf{EVAL}}$:
  $$\begin{aligned} \left| \text {Pr}\left[ {1={\textsf{Exp}}^{\textsf{sPRF},\textsf{C}}_{\mathcal {A},f,0}}\right] - \text {Pr}\left[ {1={\textsf{Exp}}^{\textsf{sPRF},\textsf{C}}_{\mathcal {A},1}}\right] \right| = \mathsf {negl(\lambda )}, \end{aligned}$$
  where ${\textsf{Exp}}^{\textsf{sPRF},\textsf{C}}_{\mathcal {A},f,0}$ and ${\textsf{Exp}}^{\textsf{sPRF},\textsf{C}}_{\mathcal {A},1}$ are defined in Fig. 7a.
- **non-adaptive PRF** [34] **in the CRS model** if for all PPT $\mathcal {A}$ which output a vector $\vec {x}$ of distinct input values *x*
  $$\begin{aligned} \left| \text {Pr}\left[ {1={\textsf{Exp}}^{\textsf{naPRF},\textsf{C}}_{\mathcal {A},f,0}}\right] - \text {Pr}\left[ {1={\textsf{Exp}}^{\textsf{naPRF},\textsf{C}}_{\mathcal {A},1}}\right] \right| = \mathsf {negl(\lambda )}, \end{aligned}$$
  where $\textsf{Exp}^{\textsf{naPRF},\textsf{crs}}_{\mathcal {A},f,0}$ and $\textsf{Exp}^{\textsf{naPRF},\textsf{crs}}_{\mathcal {A},1}$ are defined in Fig. 7b. $\textsf{C}(\lambda )=\textsf{C}$ denotes the length of the $\textsf{crs}$.
- **weak PRF** [34] if for all polynomials *N*, for all *PPT *$\mathcal {A}$
  $$\begin{aligned} \left| \text {Pr}\left[ {1={\textsf{Exp}}^{\textsf{wPRF}}_{N,\mathcal {A},f,0}}\right] - \text {Pr}\left[ {1={\textsf{Exp}}^{\textsf{wPRF}}_{N,\mathcal {A},1}}\right] \right| = \mathsf {negl(\lambda )}, \end{aligned}$$
  where $\textsf{Exp}^{\textsf{wPRF}}_{p,\mathcal {A},f,0}$ and $\textsf{Exp}^{\textsf{wPRF}}_{p,\mathcal {A},1}$ are defined in Fig. 7c.
- **pseudorandom-input PRF [this paper]** if for all polynomials *N*, for all admissible samplers $\textsf{Sam}_{\lambda ,N}$ (Definition 11) and for all *PPT* $\mathcal {A}$
  $$\begin{aligned} \left| \text {Pr}\left[ {1=\textsf{Exp}^{\mathsf {PI{\text{- }}PRF}}_{N,\mathcal {A},f,0}}\right] - \text {Pr}\left[ {1=\textsf{Exp}^{\mathsf {PI{\text{- }}PRF}}_{N,\mathcal {A},1}}\right] \right| = {negl}(\lambda ), \end{aligned}$$
  where $\textsf{Exp}^{\mathsf {PI{\text{- }}PRF}}_{N,\mathcal {A},f,0}$ and $\textsf{Exp}^{\mathsf {PI{\text{- }}PRF}}_{N,\mathcal {A},1}$ are defined as in Fig. 7d. Alternatively, if *f* satisfies the above property for a fixed $\textsf{Sam}$ (and not necessarily for arbitrary admissible sampler), we say that *f* is a $\textsf{Sam}$-PI-PRF. If the fixed $\textsf{Sam}$ is *g*-admissible, we say that *f* is $\textsf{PI}_g$-$\textsf{PRF}$.

### A conditional argument towards minimality of the definition of PI-PRF

In this section we adapt a result by Pietrzak and Sjödin [35], and show that if there exists a weak PRF that is *not* also a pseudorandom-input PRF then it can be used to build infinitely often key-agreement. Since there are wPRF candidates under assumptions which are not known to imply key-agreement, this can be seen as empirical evidence that the definition of PI-PRF we put forward is not too much of a strengthening of weak PRFs.

#### Theorem 3

(wPRF not PI-PRF implies io-KA, adapted from [35]) Let $\textsf{wPRF}$be a weak PRF. If $\textsf{wPRF}$is *not* a PI-PRF, then there exists an infinitely often correct two-party key-agreement protocol.

#### Proof

**Fig. 8:**
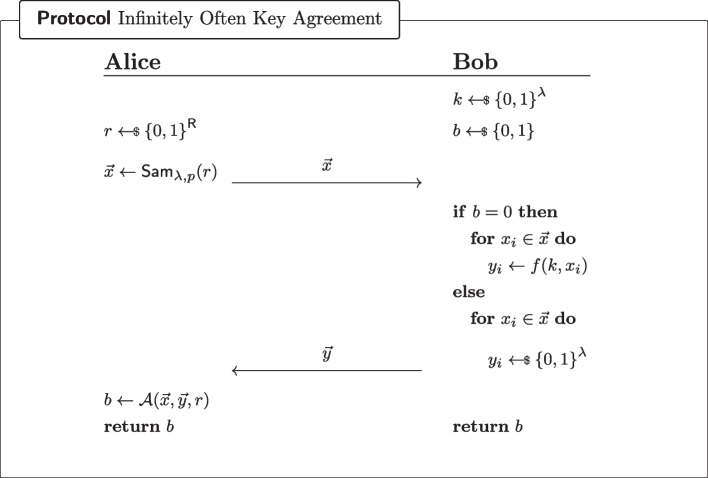
Infinitely often key-agreement protocol, assuming the existence of a weak PRF which is not a pseudorandom-input PRF

Let $\textsf{wPRF}$be a weak PRF. If $\textsf{wPRF}$is *not* a pseudorandom-input PRF, then there exists an admissible sampler $\textsf{Sam}_{\lambda ,p}$ and a $\textsf{PPT}\mathcal {A}$ with advantage $\epsilon$ in the security game of Fig. 7d (as instantiated with $\textsf{wPRF}$as the “candidate PI-PRF”). Consider the protocol of Fig. 8 (parameterised by $\mathcal {A},\textsf{Sam}_{\lambda ,p},\textsf{wPRF}$), which we will now show to be a $(\frac{1}{2}+\epsilon )$-correct single-bit infinitely often key-agreement protocol.

Correctness, *i.e.* the fact that Alice and Bob output the same bit with probability at least $(\frac{1}{2}+\epsilon )$ follows immediately from the success probability of $\mathcal {A}$ in breaking the security game of Fig. 7d. As for security, assume, for contradiction, that there is an eavesdropper $\mathcal {B}(\vec {x},\vec {y})$ that can guess *b* with probability $1/2 + \mu$ for some non-negligible $\mu$, given only the transcript of the protocol, i.e. $(\vec {x},\vec {y})$. We reach a contradiction by considering the following game hops:

- **Game 0:** Above protocol with $b = 0$
- **Game 1:** Same as Game 0, except $\vec {x}$ is sampled uniformly at random, instead of using $\textsf{Sam}$
- **Game 2:** Same as Game 1, except $b = 1$ (and hence the protocol samples $\vec {y}$ at random.)
- **Game 3:** Above protocol with $b = 1$. (Same as Game 2 but using $\textsf{Sam}$ for sampling $\vec {x}$.)

Now $\mathcal {B}$ must be able to distinguish a pair of consecutive games. However:

Game 0 is indistinguishable from Game 1 by admissibility of $\textsf{Sam}$. Game 1 is indistinguishable from Game 2 by wPRF security of *f*. Game 2 is indistinguishable from Game 3 by admissibility of $\textsf{Sam}$.

So we reach a contradiction, so such $\mathcal {B}$ cannot exist. $\square$

**Fig. 9:**
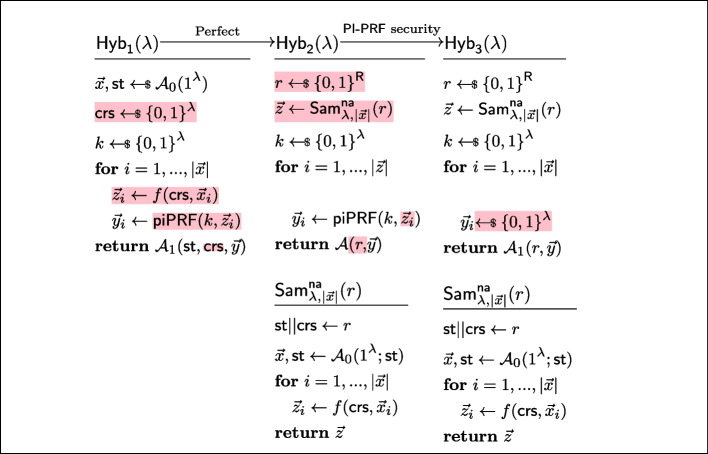
Sequence of hybrids for proving *naPRF security* in the proof of Lemma 4

### From PI-PRF to sPRF

We now provide our modular instantiation of the transformation from a PI-PRF to a sPRF, first boosting PI-PRF security via pre-processing to naPRF security and then boosting naPRF security to sPRF security via further pre-processing.

#### Lemma 4

(PI-PRF $\circ$ naPRF is a naPRF) Let *f* be a non-adaptive PRF and let $\textsf{pi}\textsf{PRF}$ be a $\mathsf {PI_f{\text{- }}PRF}$, then $\textsf{naPRF}(k,\textsf{crs},x):= \textsf{pi}\textsf{PRF}(k,f(\textsf{crs}, x))$ is a non-adaptive PRF in the CRS model.

#### Proof

Let us now show that the experiments $\textsf{Exp}^{\textsf{naPRF},\textsf{C}}_{p,\mathcal {A},f,0}$ and $\textsf{Exp}^{\textsf{naPRF},\textsf{C}}_{p,\mathcal {A},1}$ are indistinguishable by considering the hybrids $\textsf{Hyb}_1,\textsf{Hyb}_2,\textsf{Hyb}_3$ of Fig. 9.

- $\textsf{Exp}^{\textsf{naPRF},\textsf{C}}_{p,\mathcal {A},\textsf{naPRF},0}\equiv \textsf{Hyb}_1$: These hybrids are code-equivalent; we obtain $\textsf{Hyb}_1$ by inlining the definition of $\textsf{naPRF}$.
- $\textsf{Hyb}_1\equiv \textsf{Hyb}_2$: Again, these hybrids are in fact code-equivalent. Indeed, *w.l.o.g.* the second stage adversary’s state $\textsf{st}$ is equal to the first stage adversary’s internal randomness. Thus, nothing changes if we define $r:= \textsf{st}||\textsf{C}$.
- $\textsf{Hyb}_2\overset{\textrm{c}}{\approx }\textsf{Hyb}_3$: By *naPRF security* of *f*, the sampler $\textsf{Sam}^\textsf{na}_{\lambda ,|\vec {x}|}$ is *f*-admissible. The hybrids are therefore indistinguishable by $\mathsf {PI{\text{- }}PRF}$ *security* of $\textsf{pi}\textsf{PRF}$.
- $\textsf{Hyb}_3\equiv {\textsf{Exp}^{\textsf{naPRF},\textsf{C}}_{p,\mathcal {A},1}}$: These hybrids are code-equivalent (by combining the same arguments used to show the first step and $\textsf{Hyb}_1\equiv \textsf{Hyb}_2$, except in reverse).

$$\square $$

#### Lemma 5

(naPRF$\circ$ ELF is a sPRF) If $\textsf{naPRF}$ is non-adaptive PRF in the CRS model and $\textsf{ELF}$ is an extremely lossy function, then $\textsf{sPRF}(k,(\underbrace{\textsf{crs},\textsf{elf}}_{\text {new }\textsf{crs}}),x):= \textsf{naPRF}(k,\textsf{crs},\textsf{elf}(x))$ is a strong PRF in the CRS model.

Prior works [11, 12] also instantiate the non-adaptive-to-strong-PRF RO, but with a concrete hash function whose evaluation time *is a function of the adversary’s runtime*. In contrast, our construction runs in a fixed polynomial time, and is secure against general polynomial-time adversaries.

#### Proof

**Fig. 10:**
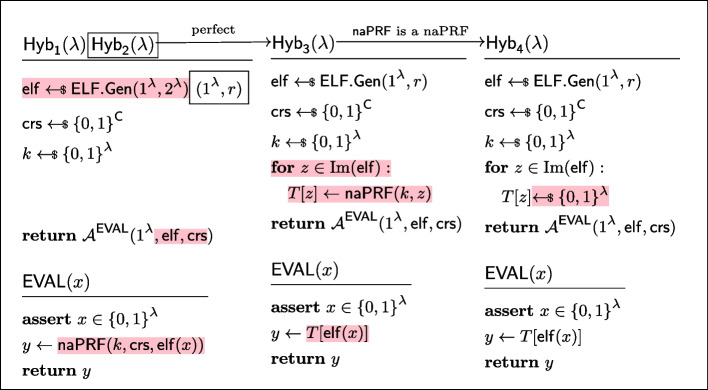
Sequence of hybrids for proving *sPRF security* in the proof of Lemma 5

We now show via several game hops (cf. Fig. 10) that the experiments ${\textsf{Exp}^{\textsf{sPRF},\textsf{C}}_{\mathcal {A},\textsf{sPRF},0}}$ and ${\textsf{Exp}^{\textsf{sPRF},\textsf{C}}_{\mathcal {A},1}}$ are indistinguishable. Assume towards contradiction, that a PPT adversary $\mathcal {A}$ has non-negligible advantage in distinguishing them. Let *r* be a sufficiently large polynomial such that $\mathcal {A}$ cannot distinguish an ELF with image size *r* from an injective ELF.

- ${\textsf{Exp}^{\textsf{sPRF},\textsf{C}}_{\mathcal {A},\textsf{sPRF},0}}\equiv \textsf{Hyb}_1$: These hybrids are code-equivalent by inlining the definition of $\textsf{sPRF}$.
- $\textsf{Hyb}_1{\overset{\textrm{c}}{\approx }}\, \textsf{Hyb}_2$: These hybrids are indistinguishable by the security of $\textsf{ELF}$.
- $\textsf{Hyb}_2\equiv \textsf{Hyb}_3$: These hybrids are code-equivalent; the only difference is pre-processing oracle calls by generating a lookup table.
- $\textsf{Hyb}_3{\overset{\textrm{c}}{\approx }}\, \textsf{Hyb}_4$: These hybrids are indistinguishable because $\textsf{naPRF}$ is a non-adaptive PRF in the CRS model. More precisely, in the $\textsf{naPRF}$ experiment, the first stage adversary $\mathcal {A}_0^\textsf{naPRF}$ samples the ELF and chooses the image of the ELF as the vector $\vec {x}$ and the description of the ELF ${\textsf{elf}}$ as the state $\textsf{st} = {\textsf{elf}}$ that is passed to the second stage adversary, in this case $\mathcal {A}_1^\textsf{naPRF}= \mathcal {A}^{\textsf{EVAL}}$. Now, since an arbitrary PPT $\mathcal {A}_1^\textsf{naPRF}$ cannot distinguish the naPRF $\textsf{naPRF}$ outputs from random, neither can $\mathcal {A}^{\textsf{EVAL}}$ who must run in time $<\!< r$ (note that arbitrary PPT adversary $\mathcal {A}_1^\textsf{naPRF}$ can emulate the $\textsf{EVAL}$ oracle calls by computing the full ELF image of size *r*).
- $\textsf{Hyb}_4{\overset{\textrm{c}}{\approx }}\, {\textsf{Exp}^{\textsf{sPRF},\textsf{C}}_{\mathcal {A},1}}$: The hybrids are equivalent, by *ELF security* of $\textsf{ELF}$, with the observation we applied the reverse of the code-equivalent transform of the first step.

$$\square $$

By combining Lemmas 4 and 5 we immediately obtain Corollary 6.

#### Corollary 6

(PI-PRF $\circ$ naPRF $\circ$ ELF is sPRF) Let $\textsf{pi}\textsf{PRF}$ be a $\mathsf {PI_f{\text{- }}PRF}$, let *f* be a non-adaptive PRF, and let $\textsf{ELF}$ be an extremely lossy function. Then

$$\begin{aligned} \textsf{sPRF}(k,(\textsf{crs},\textsf{elf}),x) := \textsf{pi}\textsf{PRF}(k, \underbrace{f(\textsf{crs},\textsf{elf}(x))}_{\text {public pre-processing}}) \end{aligned}$$

is a sPRF in the CRS model, where $\textsf{crs}{\leftarrow }{{\$}}\{0,1\}^\textsf{C}$ and $\textsf{elf}{\leftarrow }{{\$}}\textsf{ELF}.\textsf{Gen}(1^\lambda ,2^\lambda )$.

## Instantiating Hash-then-Evaluate in the distributed setting: OPRFs and PCFs

Where the hash-then-evaluate paradigm truly shines is in the distributed setting, where it allows us to only wrap the compiler of *secure multiparty computation* (which typically requires a lot more resources as the depth of computation grows), around a (low-complexity) weak PRF. This idea of applying a random oracle to the input before performing the secure evaluation of only a *weak* PRF is not merely of theoretical interest, but rather is a key ingredient in state-of-the-art OPRFs and PCFs which we now each review in turn.

### Oblivious PRFs (OPRFs)

We established in Corollary 6 that the random oracle used to transform a weak PRF to a strong one can be instantiated, provided we are willing to assume the weak PRF is in fact a *pseudorandom-input PRF*. One may pause and wonder why one would ever use this transformation given that a strong PRF can be built in a black-box way from a weak PRF, and *a fortiori* from a pseudorandom-input PRF.

An *Oblivious PRF* (OPRF) is a secure two-party protocol realising the functionality $(k,x)\mapsto (\bot ,F(k,x))$ for some pseudorandom function family *F*. If *F* is no longer assumed to be a strong PRF but instead only a weak or pseudorandom-input PRF, we will call such a protocol a *secure function evaluation (SFE) of a weak (*resp.* pseudorandom-input) PRF*.

#### Remark 1

(Defining an “Oblivious wPRF”) The problem of defining an “Oblivious weak PRF”^5^ is a delicate one, which was explicitly left open by *e.g.* [21, 30]. A first attempt would be to define it as *Secure Function Evaluation (SFE) of a weak PRF*, *i.e.* as a secure two-party protocol realising the functionality $(k,x)\mapsto (\bot ,F(k,x))$ for some *weak* pseudorandom function family *F*. This is a convenient solution from a design perspective, but it places the burden of not misusing the primitive on the user (wishing to build some larger protocol). Indeed, using such a protocol only guarantees server privacy *over the randomness of the queries* made by the client. When the primitive of *SFE of a wPRF* is composed, it becomes unclear what this means^6^; in particular, in Canetti’s *Universal Composability* framework [18] the inputs of even semi-honest parties are assumed to have been provided by a malicious environment, so even “trusting a semi-honest party to use random inputs” is not necessarily sound, unless the protocol explicitly specifies how they should be sampled. For this reason, one might argue that the ideal functionality of an *Oblivious weak PRF* should sample the queries itself, and *output* them to the client, alongside their evaluations. This definition would be analogous to those of *random OT* [36] and *random-input PIR* [27]. The downside of this alternative definition is that it does not seem possible to then use the hash-then-evaluate paradigm to boost an oblivious wPRF to an OPRF.

**Fig. 11:**
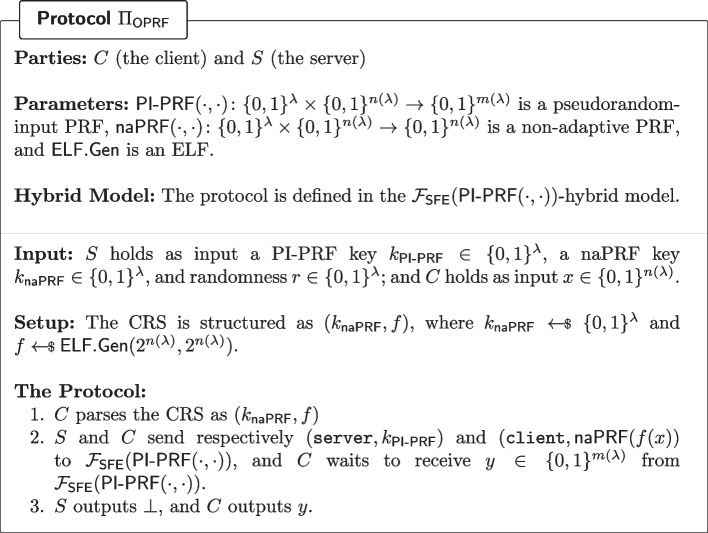
OPRF (parameterised by the PRF of Corollary 6) given secure function evaluation of a pseudorandom-input PRF

#### Lemma 7

(Hash-then-Evaluate OPRF) Let $\mathsf {PI{\text{- }}PRF}:\{0,1\}^\lambda \times \{0,1\}^{n(\lambda )}\rightarrow \{0,1\}^{m(\lambda )}$ be a pseudorandom-input PRF, let $\textsf{ELF}.\textsf{Gen}$ be an extremely lossy function, and let $\textsf{naPRF}:\{0,1\}^\lambda \times \{0,1\}^{n(\lambda )}\rightarrow \{0,1\}^{n(\lambda )}$ be a non-adaptive PRF. Then the protocol of Fig. 11 (defined in the $\mathcal {F}_\textsf{SFE}(\mathsf {PI{\text{- }}PRF})$-hybrid model, where $\mathcal {F}_\textsf{SFE}(\mathsf {PI{\text{- }}PRF}(\cdot ,\cdot ))$ is the ideal functionality computing $(k,x)\mapsto (\bot ,\textsf{wPRF}(k,x))$) is a (semi-honest) OPRF in the CRS model for the following PRF:

$$\begin{aligned} \begin{array}{rccl} \textsf{PRF}:& \{0,1\}^{3\lambda }\times \{0,1\}^{n(\lambda )}& \rightarrow & \{0,1\}^{m(\lambda )}\\ & (k=(k_\mathsf {PI{\text{- }}PRF},k_\textsf{naPRF},r),x)& \mapsto &  \mathsf {PI{\text{- }}PRF}(k_\mathsf {PI{\text{- }}PRF},\textsf{naPRF}(k_\textsf{naPRF},f(x))),\\ & & & \text {where}\, {f}=\textsf{ELF}.\textsf{Gen}(2^{n(\lambda )},2^{n(\lambda )};r) \end{array} \end{aligned}$$

#### Proof Sketch

There are two statements to prove: the first is that $\textsf{PRF}$ is a pseudorandom function family, and the second is that the Fig. 11 securely realises the functionality $(k,x)\mapsto (\bot ,\textsf{PRF}(k,x))$. We already proved the former in Corollary 6, and the latter follows immediately from the fact the only interaction between *C* and *S* is through $\mathcal {F}_\textsf{SFE}(\mathsf {PI{\text{- }}PRF}(\cdot ,\cdot ))$.

#### Remark 2

(Instantiating Sate-of-the-Art OPRF) We recall that the OPRF construction of Dinur et al. [23], using only two rounds and 641 bits of online communication, boils down to providing a special-purpose protocol for securely computing Boneh et al. s [14] weak PRF candidate. Under the assumption that this candidate is in fact a pseudorandom-input PRF (for some class of admissible samplers)—we discuss this assumption in Section —then the construction of Fig. 11 can be used to instantiate Dinur et al.’s [23] OPRF while preserving the number of rounds and the amount of communication. Depending on the desired level of security (*e.g.* malicious), some additional tools will be required.

#### Remark 3

(Removing the CRS) When considering a semi-honest adversary, the structured CRS used in the OPRF of Fig. 11 can instead be generated by the following protocol, at the cost of an additional round of interaction: (1) the client samples $k_\textsf{naPRF}$ uniformly at random, (2) client and server each sample an ELF in injective-mode, then exchange these two functions $f_{C}$ and $f_{S}$, (3) the parties proceed as in Fig. 11 but defining *f* as $f:= f_C\circ f_S$. If $f_C$ and $f_S$ are both injective, then so is *f*, but if one of them is (extremely) lossy, then so is *f* (as $|\textsf{Im}(f_C\circ f_S)|\le |\textsf{Im}(f_C)|,|\textsf{Im}(f_S)|$). This allows the reduction to switch *f* to lossy mode (even though the corrupted party samples their ELF in injective mode), and the proof goes through.

### Pseudorandom Correlation Functions (PCFs)

We introduce the notion of pseudorandom-input PCF in Section 4.2.1. In Section 4.2.2 we show a conditional argument towards the minimality of this new definition (namely, we show that if there exists a weak PCF which is not also a PI-PCF, then there is a two-party key-agreement protocol). We introduce the notion of fully non-adaptive PCF in Section 4.2.3, and then show in Section 4.2.4 that applying a non-adaptive PRF (whose key is public) to the input of a pseudorandom-input PCF yields a fully non-adaptive PCF. Finally, in Section 4.3 we show that applying an ELF to the input of a fully non-adaptive PCF yields a strong PCF.

#### Defining a Pseudorandom-Input PCF $(\mathsf {PI\text {-}PCF})$

We refer to Section 2 for a reminder on the existing notions of PCFs which have been studied in the literature: weak, non-adaptive, and strong PCFs. Of those, the one with the least constraining defintion is the *weak PCF*. Assume two parties wish to use a weak PCF for OT correlations^7^ in order to generate correlated randomness to be used for secure computation. They will need some way to agree on which OT correlations to use, *i.e.* on which points their PCF keys should be evaluated. If they were using a non-adaptive PCF, they could simply use some predetermined order, *e.g.* 1, 2, 3, *etc.*  or $(\textsf{sid},1)$, $(\textsf{sid},2)$, $(\textsf{sid},3)$, *etc.* for a session identifier $\textsf{sid}$. However, with a *weak* PCF the OT correlations will only be guaranteed to be “safe to use”^8^ when indices are chosen *uniformly* at random. Thus, the parties need to agree beforehand on a random string or a CRS which grows with the size of the computation. This raises the following question:

Is there an intermediary notion, stronger than a wPCF but weaker than a naPCF, which is directly useful for MPC applications without a CRS?

A natural idea is to replace a large random string by a *pseudorandom* string which can be generated by a pseudorandom function using a small seed which is small enough to become a part of each party’s PCF key. We thus introduce the concept of a *pseudorandom-input* PCF $(\mathsf {PI\text {-}PCF})$, which remains correct and secure even if the PCF inputs are chosen pseudorandomly, according to a *public* seed.

Again, we rely on the concept of an *admissible* sampler (Definition 11), which we previously introduced in the context of PI-PRFs.

We define a PI-PCF syntactically in the same way as a weak PCF (Definition 4), but demand the stronger properties of *pseudorandom* $\mathcal {Y}$*-correlated outputs* and *PCF security*, which we describe in Definitions 13 and 14 (differences with the corresponding notions for a weak PCF are highlighted).

##### Definition 13

**Fig. 12:**
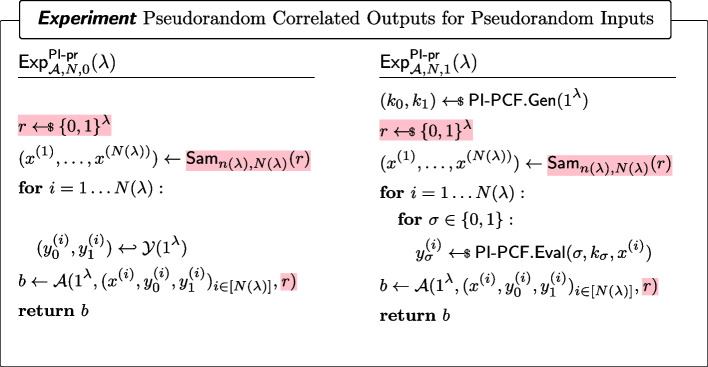
Pseudorandom $\mathcal {Y}$-correlated outputs of a PI-PCF. Differences with a wPCF (Fig. 1) are highlighted

(Pseudorandom $\mathcal {Y}$-correlated outputs of a PI-PCF) For every non-uniform PPT adversary $\mathcal {A}$, it holds that for all polynomials *N*, for all admissible samplers $\textsf{Sam}_{n(\lambda ),N}$,

$$\begin{aligned} |\Pr [\textsf{Exp}^\mathsf {PI\text {-}pr}_{\mathcal {A},N,0}(\lambda )=1]-\Pr [\textsf{Exp}^\mathsf {PI\text {-}pr}_{\mathcal {A},N,1}(\lambda )=1]| \end{aligned}$$

is negligible, where Fig. 12 defines $\textsf{Exp}^\mathsf {PI\text {-}pr}_{\mathcal {A},N,b}(\lambda )$ ($b\in \{0,1\}$).

##### Definition 14

**Fig. 13:**
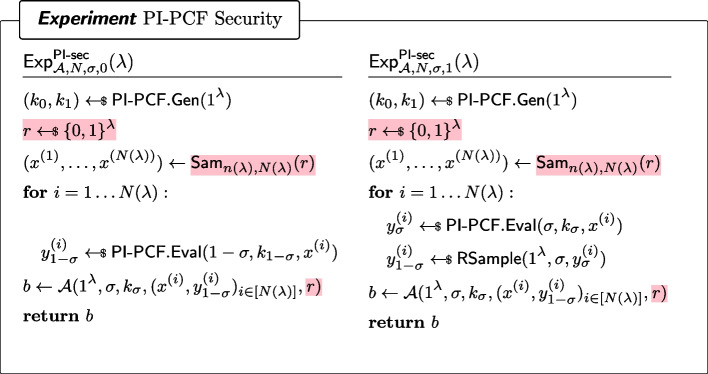
Security of a pseudorandom-input PCF. Here, $\textsf{RSample}$ is the algorithm for reverse sampling $\mathcal {Y}$ as in Definition 3

(PI-PCF Security) For every $\sigma \in \{0,1\}$ and every non-uniform PPT $\mathcal {A}$, it holds that for all polynomial *N*, for all admissible samplers $\textsf{Sam}_{n(\lambda ),N}$,

$$\begin{aligned} |\Pr [\textsf{Exp}^\mathsf {PI\text {-}sec}_{\mathcal {A},N,\sigma ,0}(\lambda )=1]-\Pr [\textsf{Exp}^\mathsf {PI\text {-}sec}_{\mathcal {A},N,\sigma ,1}(\lambda )=1]| \end{aligned}$$

is negligible, where $\textsf{Exp}^\mathsf {PI\text {-}sec}_{\mathcal {A},N,\sigma ,b}$ ($b\in \{0,1\}$) is defined as in Fig. 13.

We note that the requirement of tolerating *any* admissible sampler is a strong one (but admittedly still weaker than a non-adaptive PCF). We discuss in Remark 4 a meaningful relaxation which is still strong enough to allow our transformation to go through.

#### A conditional argument towards minimality

Let us now show that if there exists a weak PCF which is *not* a pseudorandom-input PCF, then it can be used to build an infinitely-often key-agreement scheme. This theorem is not trivial in the sense that a weak PCF can be seen as a form of *silent (and incremental) OT extension*, and is not known to imply the existence of infinitely often key-agreement^9^. Moreover, as we discuss in Section 5.4, there are plausible candidates of weak PCFs from assumptions not known to imply infinitely often key-agreement.

If $\textsf{wPCF}$ is a weak PCF for some correlation $\mathcal {Y}$, but *not* a pseudorandom-input PCF for $\mathcal {Y}$, then this means that at least one out of (1) *pseudorandom-input pseudorandom* $\mathcal {Y}$*-correlated outputs* or (2) the *pseudorandom-input PCF security* is violated. The proof proceeds by case distinction and shows that in either case, there is a key-agreement protocol with correctness at least $\frac{1}{2}+\epsilon$, for a non-negligible function $\epsilon$. The argument is very analogous to Pietrak-Sjödin [35].

##### Theorem 8

(wPCF not PI-PCF implies io-KA) Let $\textsf{wPCF}$ be a weak PCF (for some correlation). If $\textsf{wPCF}$ is *not* a pseudorandom-input PCF (for that same correlation), then there exists an infinitely often two-party key-agreement protocol.

**Fig. 14:**
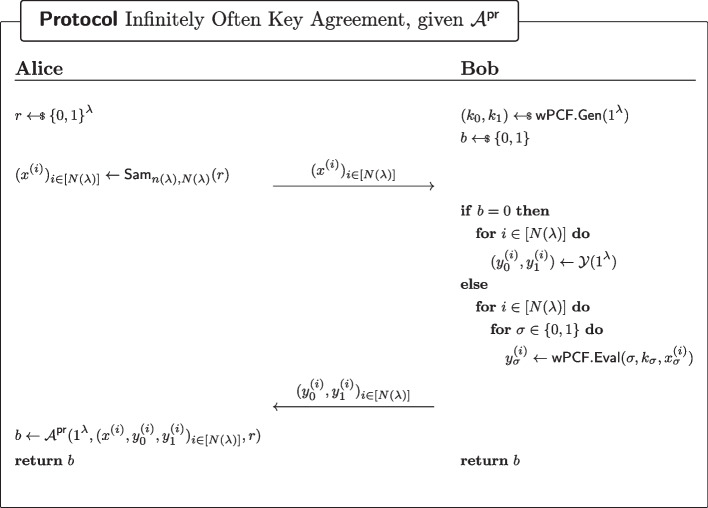
Infinitely often key-agreement scheme, assuming the existence of a wPCF which does not satisfy Definition 13

##### Proof of Theorem 8

Let $\textsf{wPCF}$ be a weak PCF for some correlation $\mathcal {Y}$. If $\textsf{wPCF}$ is *not* a pseudorandom-input PCF for $\mathcal {Y}$, then at least one of the following properties is not true: *pseudorandom-input pseudorandom* $\mathcal {Y}$*-correlated outputs* or *pseudorandom-input PCF security*. One of the following statements is therefore true:

- There exists a non-uniform polytime adversary $\mathcal {A}^\textsf{pr}$ and a non-negligible function $\epsilon (\cdot )$, such that for infinitely many $\lambda \in \mathbb {N}$, there exists a polynomial *N* and an admissible sampler $\textsf{Sam}_{n(\lambda ),N(\lambda )}$ such that:
  1 $$\begin{aligned} |\Pr [\textsf{Exp}_{\mathcal {A}^\textsf{pr},N,0}^\mathsf {PI\text {-}pr}(\lambda )=1]-\Pr [\textsf{Exp}_{\mathcal {A}^\textsf{pr},N,1}^\mathsf {PI\text {-}pr}(\lambda )=1]|> \epsilon (\lambda ) \end{aligned}$$
  where $\textsf{Exp}_{\mathcal {A}^\textsf{pr},N,b}^\mathsf {PI\text {-}pr}$ ($b\in \{0,1\}$) is defined as in Fig. 12 (but parameterised by the PCF $\textsf{wPCF}=(\textsf{wPCF}.\textsf{Gen},\textsf{wPCF}.\textsf{Eval})$).
- There exists $\sigma \in \{0,1\}$ and a non-uniform polytime adversary $\mathcal {A}^\textsf{sec}_\sigma$ and a non-negligible function $\epsilon (\cdot )$, such that for infinitely many $\lambda \in \mathbb {N}$, there exists a polynomial *N* and an admissible sampler $\textsf{Sam}_{n(\lambda ),N(\lambda )}$
  2 $$\begin{aligned} |\Pr [\textsf{Exp}^\mathsf {PI\text {-}sec}_{\mathcal {A}^\textsf{sec}_\sigma ,N,\sigma ,0}(\lambda )=1]-\Pr [\textsf{Exp}^\mathsf {PI\text {-}sec}_{\mathcal {A}^\textsf{sec}_\sigma ,N,\sigma ,1}(\lambda )=1]|> \epsilon (\lambda ) \end{aligned}$$
  where $\textsf{Exp}^\mathsf {PI\text {-}sec}_{\mathcal {A}^\textsf{sec}_\sigma ,N,\sigma ,b}$ ($b\in \{0,1\}$) is defined as in Fig. 13 (but parameterised by the PCF $\textsf{wPCF}=(\textsf{wPCF}.\textsf{Gen},\textsf{wPCF}.\textsf{Eval})$).

In either case, there is an infinitely often key-agreement protocol with correctness $\frac{1}{2}+\epsilon$.

We now show that regardless which proposition holds there exists an infinitely often key-agreement protocol with correctness $\frac{1}{2}+\epsilon$.

**Fig. 15:**
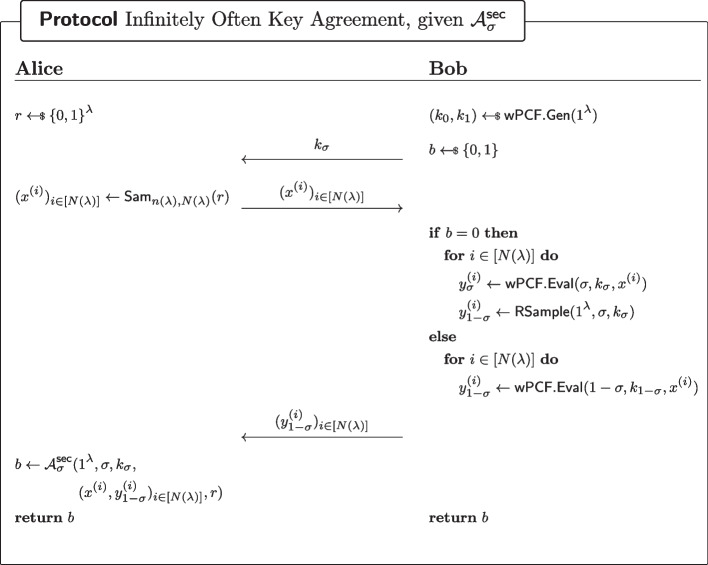
Infinitely often key-agreement scheme, assuming the existence of a wPCF which does not satisfy Definition 14

- *Given the existence of* $\mathcal {A}^\textsf{pr}$*.* Consider the protocol of Fig. 14. By (1), Alice and Bob will output the same bit with probability at least $\frac{1}{2}+\epsilon$ for infinitely many values of the security parameter, hence infinitely often correctness (recall that $\epsilon$ is non-negligible). For security consider an eavesdropper Eve with access to the transcript of the communication between Alice and Bob. Let $\lambda \in \mathbb {N}$ be a security parameter. Because the sampler $\textsf{Sam}_{n(\lambda ),N(\lambda )}$ is admissible, Eve cannot distinguish between the transcript of the real protocol, and that of a variant where Alice samples the $(x^{(i)})_{i\in [N(^\lambda )]}$ uniformly at random. In that variant however, Eve’s advantage in guessing *b* cannot be better than negligible, because the outputs of $\textsf{wPCF}$ (on uniformly random inputs $(x^{(i)})_{i\in [N(^\lambda )]}$) are pseudorandomly $\mathcal {Y}$-correlated. Hence security of the io-KA protocol.
- *Given the existence of* $\mathcal {A}^\textsf{sec}_\sigma$*, for some* $\sigma \in \{0,1\}$*.* Consider the protocol of Fig. 15. By (2), Alice and Bob will output the same bit with probability at least $\frac{1}{2}+\epsilon$ for infinitely many values of the security parameter, hence infinitely often correctness (recall that $\epsilon$ is non-negligible). For security consider an eavesdropper Eve with access to the transcript of the communication between Alice and Bob. Let $\lambda \in \mathbb {N}$ be a security parameter. Because the sampler $\textsf{Sam}_{n(\lambda ),N(\lambda )}$ is admissible, Eve cannot distinguish between the transcript of the real protocol, and that of a variant where Alice samples the $(x^{(i)})_{i\in [N(^\lambda )]}$ uniformly at random. In that variant however, Eve’s advantage in guessing *b* cannot be better than negligible, by weak PCF security of $\textsf{wPCF}$. Hence security of the io-KA protocol.

In either case, there exists an infinitely often key-agreement scheme. $\square$

#### Defining a fully non-adaptive PCF $(\textsf{fnaPCF})$.

The notion of PI-PCF we just introduced is analogous to the notion of PI-PRF we introduced in Definition 12. In Section 3.3, we showed a modular strengthening from PI-PRF to sPRF, via a naPRF; it is therefore a natural question to ask whether the same transformation can be used to first turn a PI-PCF into a naPCF, and then a naPCF into a sPCF. This would be interesting because the first transform, which does not even require the application of an ELF, has the potential of being very lightweight. Unfortunately, for technical reasons, the intermediary notion we obtain is not a non-adaptive PCF, but what we coin as a *fully non-adaptive PCF*. The difference lies in the inputs of a non-adaptive PCF must be chosen before seeing any of the evaluations of the honest party’s PCF key, but can be chosen *after* seeing the corrupt party’s PCF key.

We now introduce the notion of a *fully non-adaptive PCF (fnaPCF)*, which differs from a non-adaptive PCF in that in the PCF security game, the adversary must produce the evaluation points before even seeing the corrupt party’s PCF key. A fnaPCF is syntactically defined as a non-adaptive PCF (Definition 8) and satisfies the same notion of *non-adaptively pseudorandom* $\mathcal {Y}$*-correlated outputs* (Definition 7) as a non-adaptive PCF, but satisfies a stronger security property which we define in Definition 15 (differences with the security of a naPCF are highlighted).

##### Definition 15

**Fig. 16:**
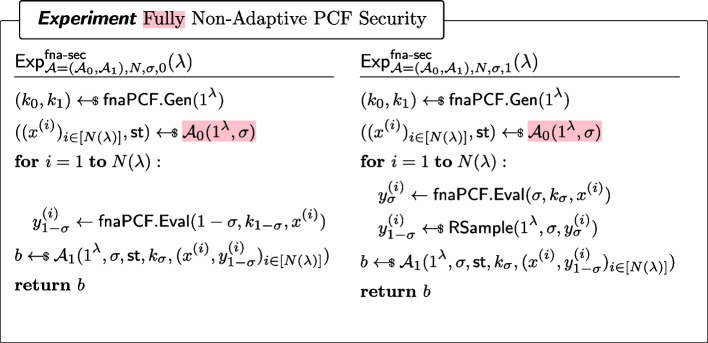
Security of a fully non-adaptive PCF. Here, $\textsf{RSample}$ is the algorithm for reverse sampling $\mathcal {Y}$ as in Definition 3. Differences with Fig. 16 are highlighted

(Fully Non-Adaptive PCF Security) For every $\sigma \in \{0,1\}$ and every non-uniform adversary $\mathcal {A}=(\mathcal {A}_0,\mathcal {A}_1)$ of size $B(\lambda )$, it holds that for all sufficiently large $\lambda$,

$$\begin{aligned} |\Pr [\textsf{Exp}^\mathsf {fna\text {-}sec}_{\mathcal {A}=(\mathcal {A}_0,\mathcal {A}_1),N,\sigma ,0}(\lambda )=1]-\Pr [\textsf{Exp}^\mathsf {fna\text {-}sec}_{\mathcal {A}=(\mathcal {A}_0,\mathcal {A}_1),N,\sigma ,1}(\lambda )=1]|\le \epsilon (\lambda ) \end{aligned}$$

where $\textsf{Exp}^\mathsf {na\text {-}sec}_{\mathcal {A}=(\mathcal {A}_0,\mathcal {A}_1),N,\sigma ,b}$ ($b\in \{0,1\}$) is defined as in Fig. 16.

#### Boosting security from $\mathsf {PI\text {-}PCF}$ to $\textsf{fnaPCF}$

Having defined the notions of pseudorandom-input and fully non-adaptive PCFs, we are ready to introduce our transform.

**Fig. 17:**
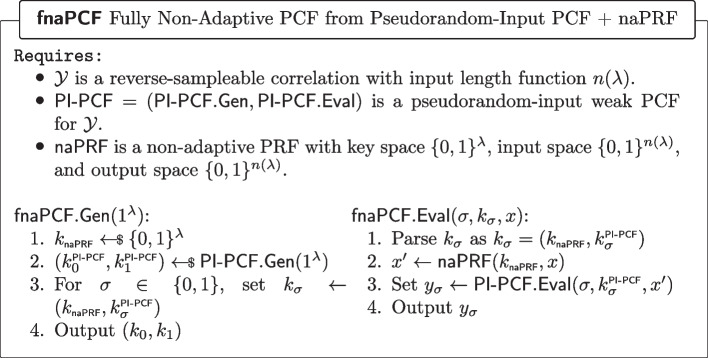
Applying a non-adaptive PRF to a pseudorandom-input PCF’s input yields a fully non-adaptive PCF

**Fig. 18:**
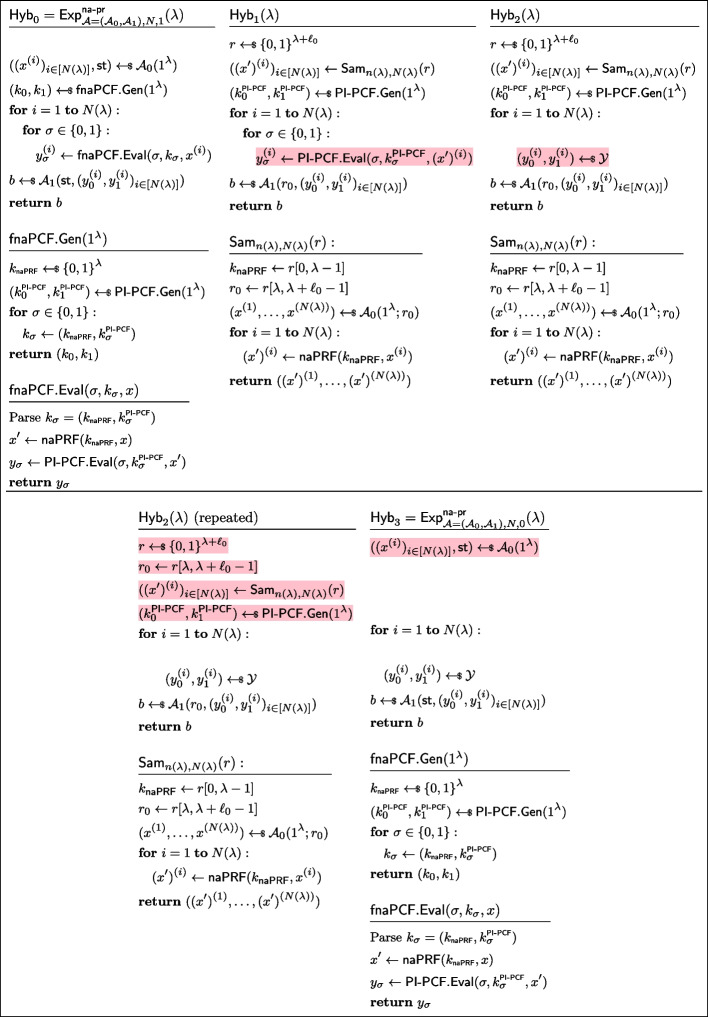
Sequence of hybrids for proving *non-adaptively pseudorandom* $\mathcal {Y}$*-correlated outputs* in the proof of Lemma 9

##### Lemma 9

(PI-PCF $\varvec{\circ }$ naPRF is a fnaPCF) Applying a non-adaptive PRF to the input of a pseudorandom-input weak PCF for some correlation $\mathcal {Y}$ yields a non-adaptive PCF for the same correlation $\mathcal {Y}$. More formally, the construction of Fig. 17 is a non-adaptive PCF.

**Fig. 19:**
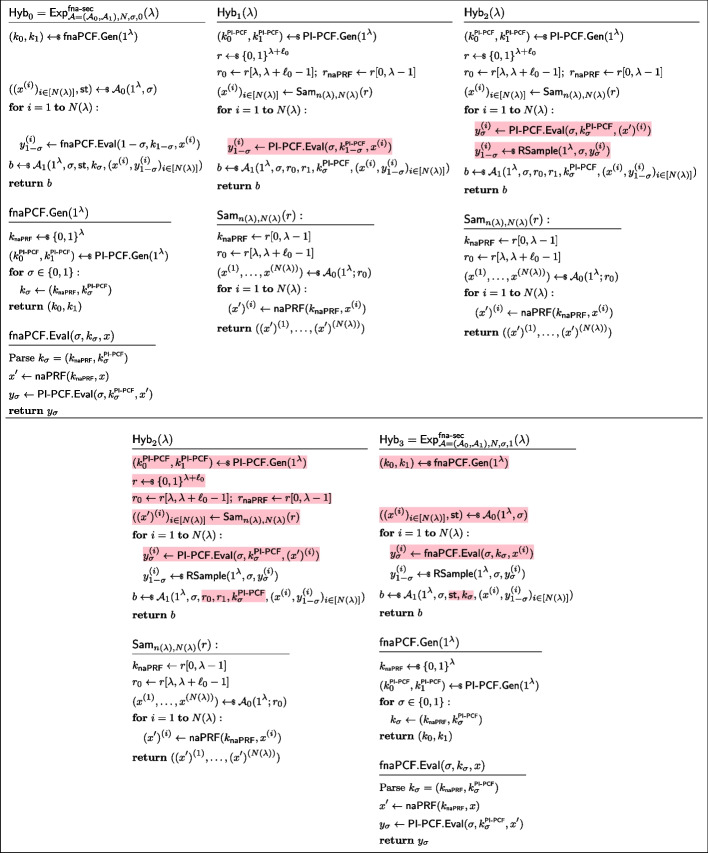
Sequence of hybrids for proving *fnaPCF security* in the proof of Lemma 9

##### Remark 4

(A PI-PCF “for all admissible samplers” is not required) By careful inspection of the proof of Lemma 9 (and specifically in the hop from hybrid $\textsf{Hyb}_1$ to $\textsf{Hyb}_2$ in Fig. 18, and in the same hop in Fig. 19), one may observe that all is required of the PI-PCF is that it tolerates admissible samplers of the form “$\textsf{Sam}_{n(\lambda ),N(\lambda )}:$ $(x^{(i)})_{i\in {[N(\lambda )]}}{{\leftarrow \!\!\!\$}\,}\mathcal {A}(1^\lambda )$; $k_\textsf{naPRF}{{\leftarrow \!\!\!{\$}\,}\,}\{0,1\}^\lambda$; For $i\in [N(\lambda )]$, $(x')^{(i)}\leftarrow \textsf{naPRF}(k_\textsf{naPRF},x^{(i)})$; Return $((x')^{(i)})_{i\in {[N(\lambda )]}}$”. This relaxation on the notion of a PI-PCF is the key to plausibly instantiating it under variants of LPN, as discussed in Section 5.4.

##### Proof of Lemma 9

Let $\mathcal {Y}$ be a reverse-sampleable correlation with input length function $n(\lambda )$, let $\mathsf {PI\text {-}PCF}=(\mathsf {PI\text {-}PCF}.\textsf{Gen},\mathsf {PI\text {-}PCF}.\textsf{Eval})$ be a pseudorandom-input weak PCF for $\mathcal {Y}$, let $\textsf{naPRF}$ be a non-adaptive PRF with key space $\{0,1\}^\lambda$, input space $\{0,1\}^{n(\lambda )}$, and output space $\{0,1\}^{n(\lambda )}$. Let $\textsf{fnaPCF}=(\textsf{fnaPCF}.\textsf{Gen},\textsf{fnaPCF}.\textsf{Eval})$ be defined as in Fig. 17.

In order to show that it is a fully non-adaptive PCF, we need to show it has *non-adaptively pseudorandom* $\mathcal {Y}$*-correlated outputs* and that it satisfies *fully non-adaptive PCF security*. Both reductions follow along the same lines as the proof of Lemma 4, showing an analogous transformation from pseudorandom-input PRF to non-adaptive PRF.

1. *Non-adaptively pseudorandom* $\mathcal {Y}$*-correlated outputs.* Let *N* be a polynomial function, and let $\mathcal {A}=(\mathcal {A}_0,\mathcal {A}_1)$ be a non-uniform adversary of size $B(\lambda )$ making at most $N(\lambda )$ non-adaptive queries. Without loss of generality, we assume that the state output by $\mathcal {A}_0$ are the random coins it used (recall that $\mathcal {A}_0$ outputs $N(\lambda )$ naPRF inputs as well as its state, to be passed to the distinguisher $\mathcal {A}_1$); we denote $\ell _0$ the length of this state/randomness. Consider the sequence of hybrids $\textsf{Hyb}_0,\textsf{Hyb}_1,\textsf{Hyb}_2,\textsf{Hyb}_3$ as defined in Fig. 18. We now show these hybrids to be indistinguishable.
  $H_0\equiv H_1$: This follows from the observation that $H_0$ and $H_1$ are code-equivalent; we simply inlined the definitions of $\textsf{fnaPCF}.\textsf{Gen}$ and $\textsf{fnaPCF}.\textsf{Eval}$, then introduced $\textsf{Sam}_{n(\lambda ),N(\lambda )}$.
  $H_1\overset{\textrm{c}}{\approx }H_2$: By security of the non-adaptive PRF $\textsf{naPRF}$, the sampler $\textsf{Sam}_{n(\lambda ),N(\lambda )}$ is admissible (Definition 11). Since the outputs of $\mathsf {PI\text {-}PCF}$are *PI-pseudorandom* $\mathcal {Y}$*-correlated* and $\textsf{Sam}_{n(\lambda ),N(\lambda )}$ is admissible, $H_1\overset{\textrm{c}}{\approx }H_2$.
  $H_2\equiv H_3$: $H_2$ and $H_3$ are code-equivalent (as the highlighted lines define random variables never subsequently used).
2. *Fully Non-adaptive PCF security.* Let $\sigma \in \{0,1\}$. Let *N* be a polynomial function, and let $\mathcal {A}=(\mathcal {A}_0,\mathcal {A}_1)$ be a non-uniform adversary of size $B(\lambda )$ making at most $N(\lambda )$ non-adaptive queries. Without loss of generality, we assume that the state output by $\mathcal {A}_0$ are the random coins it used (recall that $\mathcal {A}_0$ outputs $N(\lambda )$ naPRF inputs as well as its state, to be passed to the distinguisher $\mathcal {A}_1$); we denote $\ell _0$ the length of this state/randomness. Consider the sequence of hybrids $\textsf{Hyb}_0,\textsf{Hyb}_1,\textsf{Hyb}_2,\textsf{Hyb}_3$ as defined in Fig. 19. We now show these hybrids to be indistinguishable.
  $H_0\equiv H_1$: This follows from the observation that $H_0$ and $H_1$ are code-equivalent.
  $H_1\overset{\textrm{c}}{\approx }H_2$: By security of the non-adaptive PRF $\textsf{naPRF}$, the sampler $\textsf{Sam}_{n(\lambda ),N(\lambda )}$ is admissible (Definition 11). By applying *PI-PCF security* of $\mathsf {PI\text {-}PCF}$, with the admissible sampler $\textsf{Sam}_{n(\lambda ),N(\lambda )}$, we immediately get that $H_1\overset{\textrm{c}}{\approx }H_2$.
  $H_2\equiv H_3$: This follows from the observation that $H_2$ and $H_3$ are code-equivalent (Completely analogously to how $H_1$ and $H_1$, as these games use the same primitives of $\textsf{Sam}_{n(\lambda ),N(\lambda )}$, $\textsf{fnaPCF}.\textsf{Gen}$, and $\textsf{fnaPCF}.\textsf{Eval}$). $\square$

### Boosting security from $\textsf{fnaPCF}$to $\textsf{sPCF}$

We now show a transform from a fully non-adaptive to a strong PCF.

**Fig. 20:**
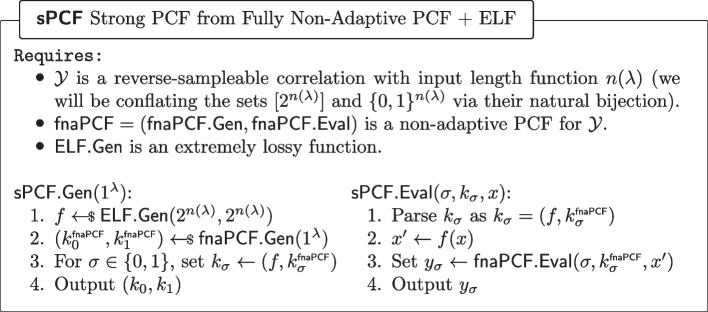
Fully non-adaptive PCF + ELF yields a strong PCF

#### Lemma 10

($\text {fnaPCF}\varvec{\circ }\text {ELF}$ is a $\text {sPCF}$)**.** Applying an ELF to the input of a fully non-adaptive PCF for some correlation $\mathcal {Y}$ yields a strong PCF for the same correlation $\mathcal {Y}$. More formally, the construction of Fig. 20 is a strong PCF.

#### Proof

Let $\mathcal {Y}$ be a reverse-sampleable correlation with input length function $n(\lambda )$, let $\mathsf {\textsf{fnaPCF}}=(\mathsf {\textsf{fnaPCF}}.\textsf{Gen},\mathsf {\textsf{fnaPCF}}.\textsf{Eval})$ be a pseudorandom-input weak PCF for $\mathcal {Y}$, let $\textsf{ELF}.\textsf{Gen}$ be an extremely lossy function. Let $\textsf{sPCF}=(\textsf{sPCF}.\textsf{Gen},\textsf{sPCF}.\textsf{Eval})$ be defined as in Fig. 20.

In order to show that it is a strong PCF, we need to show it has *strongly pseudorandom* $\mathcal {Y}$*-correlated outputs* and that it satisfies *strong PCF security*. Both reductions follow along the same lines as the proof of Lemma 5, showing an analogous transformation from non-adaptive PRF to strong PRF.

**Fig. 21:**
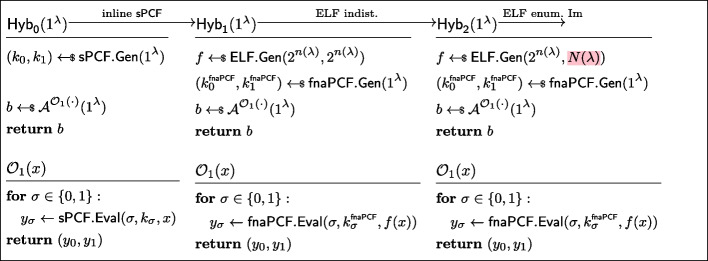
Sequence of hybrids for proving *strongly pseudorandom* $\mathcal {Y}$*-correlated outputs* in the proof of Lemma 10 (Part 1/3). $\textsf{Hyb}_0=\textsf{Exp}^\mathsf {s\text {-}pr}_{\mathcal {A},1}(\lambda )$

**Fig. 22:**
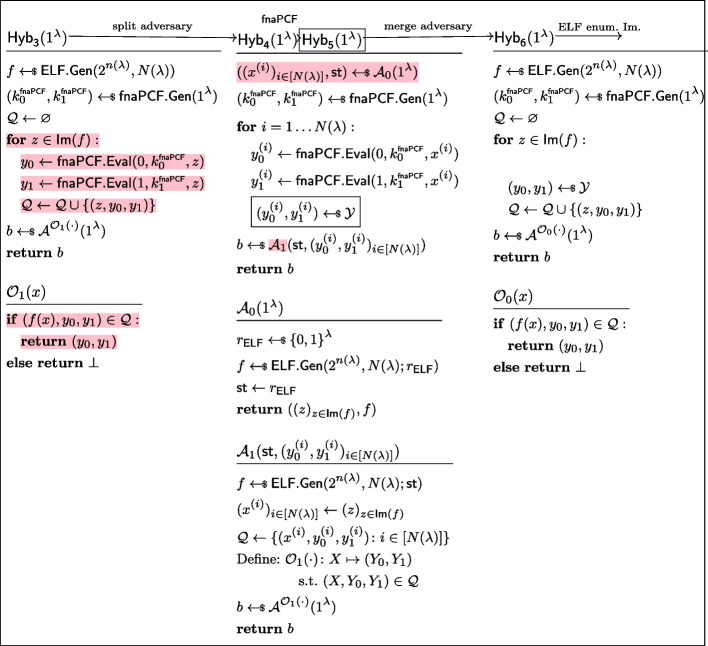
Sequence of hybrids for proving *strongly pseudorandom* $\mathcal {Y}$*-correlated outputs* in the proof of Lemma 10 (Part 2/3)

1. *Strongly pseudorandom* $\mathcal {Y}$*-correlated outputs.* Let $\mathcal {A}$ be an non-uniform adversary of size $B(\lambda )$ asking at most $N(\lambda )$ queries to the oracle $\mathcal {O}_b(\cdot )$ (as defined in Fig. 5) Consider the sequence of hybrids $(\textsf{Hyb}_i)_{i\in [0,9]}$, as defined in Fig. 21, 22, and 23. Let us now show these hybrids to be instinguishable.
  $\textsf{Hyb}_0\equiv \textsf{Hyb}_1$: These hybrids are code equivalent, we simply “inlined” the codes of $\textsf{sPCF}.\textsf{Gen}$ and $\textsf{sPCF}.\textsf{Eval}$.
  $\textsf{Hyb}_1\overset{\textrm{c}}{\approx }\textsf{Hyb}_2$: These hybrids are indistinguishable by security of $\textsf{ELF}$. Indeed otherwise, the PPT process of running $\textsf{fnaPCF}.\textsf{Gen}$, emulating $\mathcal {O}_1$, and running $\mathcal {A}^{\mathcal {O}_1(\cdot )}$ would constitute an efficient distinguisher for the ELF security game.
  $\textsf{Hyb}_2\equiv \textsf{Hyb}_3$: These hybrids are code-equivalent; observe that we simply moved the brunt of the work of $\mathcal {O}_1$ to a pre-processing phase inside $\textsf{Hyb}_3$.
  $\textsf{Hyb}_3\equiv \textsf{Hyb}_4$: These hybrids are code-equivalent; we simply reorganised the code to introduce $\mathcal {A}_0$ and $\mathcal {A}_1$.
  $\textsf{Hyb}_4\overset{\textrm{c}}{\approx }\textsf{Hyb}_5$: These hybrids are indistinguishable by the property of *non-adaptively pseudorandom* $\mathcal {Y}$*-correlated outputs* of $\textsf{fnaPCF}$.
  $\textsf{Hyb}_5\equiv \textsf{H}_6$: These hybrids are code equivalent (this hop is essentially the reverse of the transformation from $\textsf{Hyb}_3$ to $\textsf{Hyb}_4$).
  $\textsf{Hyb}_6\equiv \textsf{Hyb}_7$: These hybrids are code equivalent (this hop is essentially the reverse of the transformation from $\textsf{Hyb}_2$ to $\textsf{Hyb}_3$).
  $\textsf{Hyb}_7\overset{\textrm{c}}{\approx }\textsf{Hyb}_8$: These hybrids are indistinguishable by security of $\textsf{ELF}$(with the exact same argument used to show $\textsf{Hyb}_1\overset{\textrm{c}}{\approx }\textsf{Hyb}_2$).
  $\textsf{Hyb}_8\equiv \textsf{Hyb}_9$: These hybrids are code equivalent (this hop is essentially the reverse of the transformation from $\textsf{Hyb}_0$ to $\textsf{Hyb}_1$).
2. *Strong PCF security.* Let $\sigma \in \{0,1\}$. Let $\mathcal {A}$ be an non-uniform adversary of size $B(\lambda )$ asking at most $N(\lambda )$ queries to the oracle $\mathcal {O}_b(\cdot )$ (as defined in Fig. 6). Consider the sequence of hybrids $(\textsf{Hyb}_i)_{i\in [0,9]}$, as defined in Figs. 24, 25, and 26. Let us now show these hybrids to be instinguishable.
  $\textsf{Hyb}_0\equiv \textsf{Hyb}_1$: These hybrids are code equivalent, we simply “inlined” the codes of $\textsf{sPCF}.\textsf{Gen}$ and $\textsf{sPCF}.\textsf{Eval}$.
  $\textsf{Hyb}_1\overset{\textrm{c}}{\approx }\textsf{Hyb}_2$: These hybrids are indistinguishable by security of $\textsf{ELF}$. Indeed otherwise, the PPT process of running $\textsf{fnaPCF}.\textsf{Gen}$, emulating $\mathcal {O}_1$, and running $\mathcal {A}^{\mathcal {O}_1(\cdot )}$ would constitute an efficient distinguisher for the ELF security game.
  $\textsf{Hyb}_2\equiv \textsf{Hyb}_3$: These hybrids are code-equivalent; observe that we simply moved the brunt of the work of $\mathcal {O}_1$ to a pre-processing phase inside $\textsf{Hyb}_3$.
  $\textsf{Hyb}_3\equiv \textsf{Hyb}_4$: These hybrids are code-equivalent; we simply reorganised the code to introduce $\mathcal {A}_0$ and $\mathcal {A}_1$.
  $\textsf{Hyb}_4\overset{\textrm{c}}{\approx }\textsf{Hyb}_5$: These hybrids are indistinguishable by the property of *fully non-adaptive PCF security* of $\textsf{fnaPCF}$.
  $\textsf{Hyb}_5\equiv \textsf{Hyb}_6$: These hybrids are code equivalent (this hop is essentially the reverse of the transformation from $\textsf{Hyb}_3$ to $\textsf{Hyb}_4$).
  $\textsf{Hyb}_6\equiv \textsf{Hyb}_7$: These hybrids are code equivalent (this hop is essentially the reverse of the transformation from $\textsf{Hyb}_2$ to $\textsf{Hyb}_3$).
  $\textsf{Hyb}_7\overset{\textrm{c}}{\approx }\textsf{Hyb}_8$: These hybrids are indistinguishable by security of $\textsf{ELF}$(with the exact same argument used to show $\textsf{Hyb}_1\overset{\textrm{c}}{\approx }\textsf{Hyb}_2$).
  $\textsf{Hyb}_8\equiv \textsf{Hyb}_9$: These hybrids are code equivalent (this hop is essentially the reverse of the transformation from $\textsf{Hyb}_0$ to $\textsf{Hyb}_1$). $\square$

**Fig. 23:**
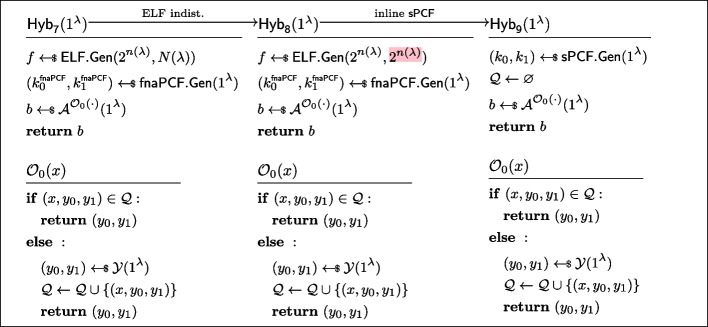
Sequence of hybrids for proving *strongly pseudorandom* $\mathcal {Y}$*-correlated outputs* in the proof of Lemma 10 (Part 3/3). $\textsf{Hyb}_9=\textsf{Exp}^\mathsf {s\text {-}pr}_{\mathcal {A},0}(\lambda )$

By combining Lemmas 9 and 10 we immediately get Corollary 11.

#### Corollary 11

(PI-PCF $\varvec{\circ }$ naPRF $\varvec{\circ }$ ELF is a sPCF) Applying an ELF then a non-adaptive PRF to the input of a pseudorandom-input weak PCF for some correlation $\mathcal {Y}$ yields a strong PCF for the same correlation $\mathcal {Y}$.

**Fig. 24:**
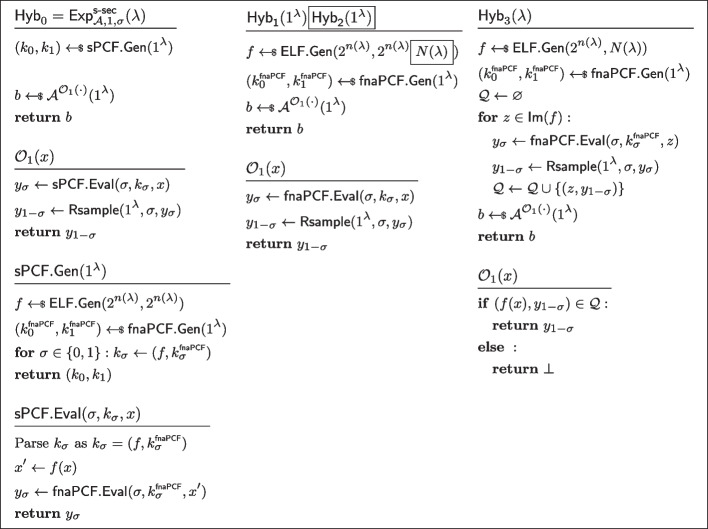
Sequence of hybrids for proving *strong PCF security* in the proof of Lemma 10 (Part 1/3)

**Fig. 25:**
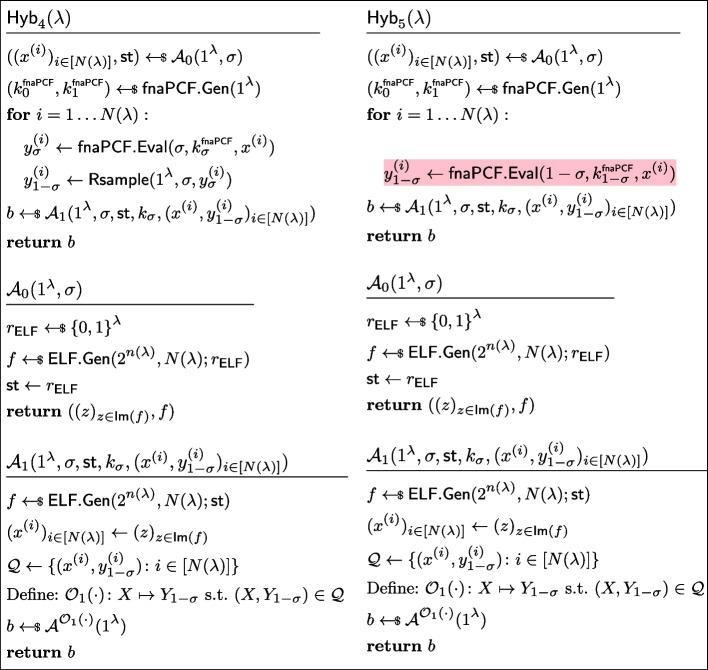
Sequence of hybrids for proving *strong PCF security* in the proof of Lemma 10 (Part 2/3)

**Fig. 26:**
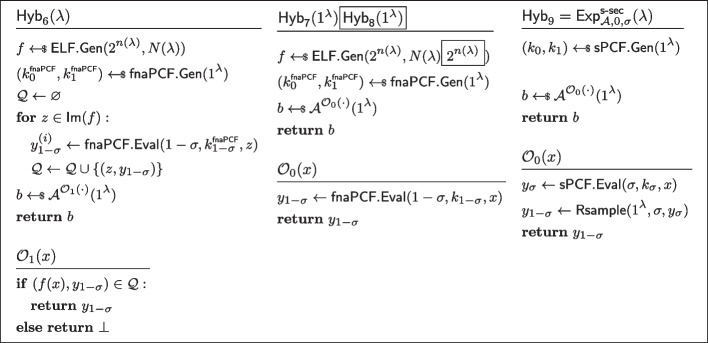
Sequence of hybrids for proving *strong PCF security* in the proof of Lemma 10 (Part 3/3)

## Candidate PI-PRFs and PI-PCFs

Our work introduces PI-PRFs as a strengthening of wPRFs. In this section, we study several wPRF candidates, which are at the heart of some of the most efficient OPRFs and the most efficient PCFs known to date. We first overview the candidates that will be analyzed in this section, and provide some motivation for their study.

### Overview of the candidates

#### First candidate: BIPSW1

A very efficient wPRF candidate is $F_K(x) = \textsf{map}(K\cdot x)$, where *K* is a matrix, *x* is a vector, $K\cdot x$ denotes matrix-vector multiplication, and $\textsf{map}$ is some fixed mapping which, on input a vector *y*, returns $\sum _i [y_i \mod 2] \bmod 3$ [14]:

##### Candidate 1

Given a matrix $K \in \mathbb {F}_2^{n\times n}$ as a key and an input vector $x\in \mathbb {F}_2^n$, $F_K(x) := \textsf{map}(K\cdot x)$ where $\textsf{map}(y) := \sum _{i\le n} [y_i \bmod 2] \bmod 3$.

We note that in their work, the authors consider the more general case $K \in \mathbb {F}_2^{m\times n}$, but use $m=n$ as their suggested instantiation. The analysis extends easily to other values of *m*.

##### Motivation

Candidate 1 is at the heart of the most efficient (to date) candidate post-quantum oblivious PRF protocol [23].

#### Second candidate: BIPSW2

The same work [14] also introduces an alternative wPRF candidate that has the downside of being broken in subexponential time using a BKW-style attack (in contrast, Candidate 1 is plausibly *exponentially* secure). On the plus side, this candidate returns elements of $\mathbb {F}_2$, which is typically more convenient than outputs over $\mathbb {F}_3$.

##### Candidate 2

Given a vector $k\in \mathbb {F}_2^n$ as a key and an input vector $x\in \mathbb {F}_2^n$, $F_k(x) := \lceil \langle k, x\rangle \bmod 6 \rfloor _2$ where for any $y \in \mathbb {Z}_6$, $\lceil y \rfloor _2 := 0$ if $y\in \{0,1,2\}$ and 1 else.

##### Motivation

Candidate 2 was recently used in a very efficient construction of OPRF [2] that achieves several appealing properties (such as partial obliviousness and heuristically yieldinh a *verifiable* OPRF) with unmatched efficiency. It leverages the compatibility between Candidate 2 and torus-FHE. In addition, Candidate 2 was recently used in the first construction of efficient public-key PCF for OT [8] and in a very fast PCF for the list-OT correlation in [20].

#### Third candidate: VDLPN

In the following, $N = 2^{D}$ is a bound on the maximum number of samples that an adversary can obtain (where $D= D(\lambda )$ is polynomial in the security parameter). Fix parameters $\textsf{par} = (\lambda ,D, N = 2^D)$. Let $\mathcal {R}_{\lambda ,i}$ be the distribution of random $\lambda$-regular vectors over $\mathbb {F}_2^{\lambda \cdot 2^i}$, i.e., a sample from $\mathcal {R}_{\lambda ,i}$ is obtained by concatenating $\lambda$ independent length-$2^i$ unit vectors. We let $\mathcal {H}_{\textsf{vd}}^i(\textsf{par})$ denote the distribution over $N\times (\lambda \cdot 2^i)$ matrices over $\mathbb {F}_2$ where each row is sampled independently from $\mathcal {R}_{\lambda ,i}$, and $\mathcal {H}_\textsf{vd}({\textsf{par}})$ denote the distribution over $\mathbb {F}_2^{N\times 2N}$ obtained by sampling $H_i \leftarrow \!\!\!{\$}\,\,\mathcal {H}_\textsf{vd}^i(\textsf{par})$ for $i=1$ to $D$ and outputting $H = H_1 || \cdots || H_D$. Eventually, we denote by $\mathcal {N}_\textsf{vd}({\textsf{par}})$ the noise distribution obtained by sampling $\textbf{e}^\intercal _i {\leftarrow \!\!\!{\$}\,}\, \mathcal {R}_{\lambda ,i}$ and outputting $\textbf{e} \leftarrow (\textbf{e}_1 /\hspace{-.08cm}/\cdots /\hspace{-.08cm}/\textbf{e}_D) \in \mathbb {F}_2^{2N}$ (that is, $\textbf{e}^\intercal$ is distributed as a row of *H*).

##### Definition 16

($\textsf{VDLPN}(\lambda ,D,N)$) The *variable-density learning parity with noise* assumption with sparsity $\lambda$, $D$ blocks, and number of samples *N*, denoted $\textsf{VDLPN}(\lambda ,D,N)$, states that

$$\begin{aligned}&\{(H,\textbf{b})\, | \,H\leftarrow \!\!\!{\$}\,\mathcal {H}_\textsf{vd}({\textsf{par}}), \textbf{e}\leftarrow \!\!\!{\$}\,\mathcal {N}_\textsf{vd}({\textsf{par}}), \textbf{b} \leftarrow H\cdot \textbf{e}\}\\ \overset{\text {c}}{\approx }&\{(H,\textbf{b}) \,|\, H\leftarrow \!\!\!{\$}\,\mathcal {H}_\textsf{vd}({\textsf{par}}), \textbf{b} \leftarrow \!\!\!{\$}\,\mathbb {F}_2^N\}. \end{aligned}$$

The VDLPN assumption parametrized with any $D= \textsf{poly}$ immediately yields a wPRF *F*:

##### Candidate 3

The vector $\textbf{e} \leftarrow \!\!\!{\$}\,\mathcal {N}_\textsf{vd}(\textsf{par})$ defines the secret key of *F*. On input a random $x \leftarrow \!\!\!{\$}\,\{0,1\}^{\lambda \cdot \sum _{i\le D} i}$, parse *x* into $D$ blocks $x_i$ of $\lambda \cdot i$ bits, each divided into $\lambda$ strings $x_{i,j} \in \{0,1\}^i$. Map each $x_{i,j}$ to the length-$2^i$ unit vector which has a 1 at $x_{i,j}$. Let $\textsf{map}(x)$ denote the concatenation of all these unit vectors. Output $F_{\textbf{e}}(x) = \textsf{map}(x)^\intercal \cdot \textbf{e}$.

For a random *x*, by construction, $\textsf{map}(x)$ is equally distributed to sampling a uniformly random column of *H*. Therefore, breaking the security of the above wPRF after receiving *N* samples is equivalent to breaking the VDLPN assumption.

##### Motivation

The VDLPN wPRF is at the heart of the PCF introduced in [5], that introduced the first PCF (for the VOLE and OT correlations). A recent variant of this candidate was introduced in [19]. An analogous security analysis applies to this variant, and the variant is shown to provide a competitive PCF candidate.

#### Fourth candidate: EALPN

Fix parameters $\textsf{par} = (\lambda , c, N)$. *N* is the number of samples, *c* is a matrix sparsity parameter (typically $c = \Theta (\log N)$ or $\omega (\log N)$), and $\lambda$ is the Hamming weight of the noise. Let $\Delta _N$ denote a 5*N*-by-5*N* lower triangular matrix filled with ones. We let $\mathcal {H}_\textsf{ea}(\textsf{par})$ denote the distribution obtained by sampling an *N*-by-5*N* matrix *M* whose entries are independent Bernoulli sample equal to 1 with probability *c*/5*N*, and outputting $H = M\cdot \Delta _N$. We denote by $\mathcal {N}_\textsf{ea}(\textsf{par})$ the distribution obtained by concatenating $\lambda$ random unit vectors of length $5N/\lambda$.

##### Definition 17

($\textsf{EALPN}(\lambda ,c,N)$) The *expand-accumulate learning parity with noise* assumption with noise weight $\lambda$, matrix sparsity *c*, and number of samples *N*, denoted $\textsf{EALPN}(\lambda ,c,N)$, states that

$$\begin{aligned}&\{(H,\textbf{b})\, |\, H\leftarrow \!\!\!{\$}\,\mathcal {H}_\textsf{ea}({\textsf{par}}), \textbf{e}\leftarrow \!\!\!{\$}\,\mathcal {N}_\textsf{ea}({\textsf{par}}), \textbf{b} \leftarrow H\cdot \textbf{e}\}\\ {\mathop {\approx }\limits ^{\text {c}}}&\{(H,\textbf{b}) \,| \,H\leftarrow \!\!\!{\$}\,\mathcal {H}_\textsf{ea}({\textsf{par}}), \textbf{b} \leftarrow \!\!\!{\$}\,\mathbb {F}_2^N\}. \end{aligned}$$

##### Candidate 4

The vector $\textbf{e}$ defines the secret key of *F*. The random input *x* is used to sample a row $M_x$ of the matrix *M* (where each entry is equal to 1 with probability *c*/5*N*). The function $F_{\textbf{e}}(x)$ outputs $M_x\cdot \Delta _N\cdot \textbf{e}$.

##### Motivation

The EALPN wPRF is at the heart of the PCF introduced in [7], which is the most efficient PCF to date.

### Attack frameworks

For each of the candidates, we analyze natural families of attacks against their security:

**Statistical query attacks** The complexity of BIPSW1 lies just barely above $\textsf{AC}^0$, a complexity class which cannot contain wPRFs (with better than quasi-polynomial security) due to *statistical query attacks* [31]. [14] show that their BIPSW1 withstands statistical query attacks as a wPRF, and mention in passing that the analysis extends analogously to their alternative candidate BIPSW2. We strengthen these results by showing afterwards that both BIPSW1 and BIPSW2 can be shown to withstands statistical query attacks as PI-PRFs as well.

**Linear tests** The VDLPN and EALPN wPRFs at the heart of the leading PCF candidates in [5] and [7] are both are LPN-style constructions. As in previous works, we analyze their security against attacks from the *linear test framework*, which captures most known attacks on the LPN assumption and its many variants.

Assuming the security of the underlying non-adaptive PRF, we establish win-win results for VDLPN and EALPN: if one finds a linear attack against the assumption that these candidates are PI-PRFs then this would have surprising consequences.

Our analysis provides support for the notion of PI-PRF, showing that natural wPRF candidates used in leading applications are plausibly also PI-PRF. Finally, we note that all of the above candidates are provably *not* strong PRFs.

### Security of BIPSW1 and BIPSW2 against statistical query attacks

To support the security of their candidate, one of the main arguments used by the authors of [14] is that with high probability over *k*, the function $F_k$ does not correlate with any fixed sufficiently small function family. This implies that their candidate cannot be broken by *statistical query algorithms* [1]. The lack of correlation of BIPSW1 with any sufficiently small function family is formalized as follows:

#### Lemma 12

([14]) Let $\mathcal {H} = \{h: \{0,1\}^\lambda \mapsto \{-1,0,1\}\}$ be a collection of functions of size *s*. Then

$$\begin{aligned} \Pr _{k{\leftarrow }{{\$}}\mathbb {F}_2^{\lambda \times \lambda }}\left[ \exists h\in \mathcal {H}\;\left| \; \Pr _x[\textsf{map}(k\cdot x) = h(x)]> \frac{1}{3} + \frac{1}{2^{\lambda -1}} + \varepsilon \right. \right] \le \frac{5s}{2^\lambda \cdot \varepsilon ^2}. \end{aligned}$$

Note that the factor $\tfrac{1}{3}$ appears in the above inequality, since the outputs of the wPRF are over $\{-1,0,1\}$ rather than $\{0,1\}$. We formulate the analogeous statement for BIPSW2 (the statement and the proof are absent from [14] but can be derived easily by adapting their analysis of BIPSW1):

#### Lemma 13

Let $\mathcal {H}= \{h: \{0,1\}^\lambda \mapsto \{0,1\}\}$ be a collection of functions of size *s*. Then

$$\begin{aligned} \Pr _{k{\leftarrow }{{\$}}\mathbb {F}_2^\lambda }\left[ \exists h\in \mathcal {H}\;\left| \; \Pr _x[\lceil \langle k, x\rangle \bmod 6 \rfloor _2 = h(x)]> \frac{1}{2} + \varepsilon \right. \right] \le \frac{5s}{2^\lambda \cdot \varepsilon ^2}. \end{aligned}$$

Lemmas 12 and 13 refer to a probability over *uniformly random* inputs *x* to the function, and thus, are only meaningful when the function is used as a wPRF. In turn, we are interested in the setting where the inputs are given by an admissible sampler $\textsf{Sam}$ that returns *pseudorandom* inputs. We show that both BIPSW1 and BIPSW2 are in fact also immunised against all correlation attacks against their PI-PRF security. In the theorems below, we consider a sampler $\textsf{Sam}(r)$ with randomness length $\ell$ and index space $\{1 \dots p\}$ that outputs $(x_1, \cdots , x_q)$ and write $\textsf{Sam}_i(r)$ for the function that returns $x_i$.

#### Theorem 14

Let $\mathcal {H}= \{h: \{0,1\}^\ell \times \{1 \dots p\} \mapsto \{-1,0,1\}\}$ be a collection of functions of size *s*. Then there exists a negligible function $\mathsf {negl(\lambda )}$ such that

$$\begin{aligned} \Pr _k\left[ \exists h\in \mathcal {H}, \Pr _{r,i}[\textsf{map}(k\cdot \textsf{Sam}_i(r)) = h(r,i)] > \frac{1}{3} + \mathsf {negl(\lambda )}+ \varepsilon \right] \le \frac{s}{\varepsilon ^2}\cdot \mathsf {negl(\lambda )}. \end{aligned}$$

#### Theorem 15

Let $\mathcal {H}= \{h: \{0,1\}^\ell \times \{1 \dots p\} \mapsto \{0,1\}\}$ be a collection of functions of size *s*. Then there exists a negligible function $\mathsf {negl(\lambda )}$ such that

$$\begin{aligned} \Pr _k\left[ \exists h\in \mathcal {H}, \Pr _{r,i}[\lceil \langle k, \textsf{Sam}_i(r)\rangle \bmod 6 \rfloor _2 = h(r,i)] > \frac{1}{2} + \varepsilon \right] \le \frac{s}{\varepsilon ^2}\cdot \mathsf {negl(\lambda )}. \end{aligned}$$

In the above theorems, the inner probabilities are over the random choice of the randomness *r* of the sampler, and of the index *i* of the sampler output. These theorems imply that the PI-PRF security of the candidate of [14] cannot be broken by statistical query analysis, an important class of attacks against wPRFs. In particular, this captures the attack of Linial, Mansour, and Nisan [31] which breaks all candidates wPRFs in $\textsf{AC}^0$ in quasipolynomial time.

#### Remark

The term $\mathsf {negl(\lambda )}$ in Theorems 14 and 15 directly come from the negligible bound on the probability that any polynomial-time adversary distinguishes the sampler output from random. Stronger assumptions on the admissible sampler, such as subexponential or exponential pseudorandomness, directly translate to a corresponding smaller $\mathsf {negl(\lambda )}$ term in Theorems 14 and 15.

#### Proof

Let $\textsf{Sam} = \textsf{Sam}_{\lambda ,p}: \{0,1\}^\ell \mapsto (\{0,1\}^\lambda )^p$ denote an admissible sampler. Fix any $i \le p$ and let $S_i$ denote $\textsf{Sam}_i^{-1}(0^\lambda ):= \{r \in \{0,1\}^\ell :\; \textsf{Sam}_i(r) = 0^\lambda \}$.

#### Claim 1

For any $i\le p$, there exists a negligible function $\mathsf {negl(\lambda )}$ such that $|S_i|/2^\ell = \mathsf {negl(\lambda )}$.

#### Proof

Assume towards contradiction that there exists $i \le p$ and a polynomial $q(\lambda )$ such that $|S_i|/2^\ell \ge 1/q(\lambda )$. Let $\mathcal {A}$ denote the following adversary against the pseudorandomness of $\textsf{Sam}$: given a tuple $(x_1, \cdots , x_p)$, $\mathcal {A}$ outputs 1 if $x_i = 0$, and returns a uniformly random bit otherwise. Observe that

$$\begin{aligned}&\left| \text {Pr}_{r {\leftarrow \!\!\$}\, \{0,1\}^{\ell }}\left[ {1=\mathcal {A}(\textsf{Sam}_{\lambda ,p}(r))}\right] - \text {Pr}_{\forall i: x_{i} {\leftarrow \!\!\$}\, \{0,1\}^\lambda }\left[ {1=\mathcal {A}(x_1,\cdots ,x_p)}\right] \right| \\&\ge \left| \frac{1}{q(\lambda )} - \frac{1}{2^\lambda }\right|> \frac{1}{2q(\lambda )}, \end{aligned}$$

which contradicts the assumption that $\textsf{Sam}$ is an admissible sampler. $\square$

Let $T_M \leftarrow \max _{i\le p} |\textsf{Sam}_i^{-1}(0^\lambda )|$ and $T_m \leftarrow \min _{i\le p} |\textsf{Sam}_i^{-1}(0^\lambda )|$. Note that by the above claim, $T_m/2^\ell$ and $T_M/2^\ell$ are both negligible in $\lambda$. The rest of the proof largely follows the analysis of [14], and adapts it to our setting. Along the way, we also fix a minor bug in the original analysis (we notified the authors). Let $\mathbbm {1}(a,b)$ denote the indicator function which outputs 1 iff $a=b$, and 0 otherwise.

In the following, we let $F_k(x)$ denote either BIPSW1 or BIPSW2 (*k* denotes a matrix for BIPSW1 and a vector for BIPSW2, but we do not distinguish for simplicity) and only specify a concrete candidate when the proof differs. Fix a single $h\in \mathcal {H}$; then, we will conclude with a union bound over all elements of $\mathcal {H}$.

First, we consider the following expectation, which we bound in both directions:

$$\begin{aligned}&\mathbb {E}_k\left[ \Pr _{r,i}[F_k(\textsf{Sam}_i(r)) = h(r,i)]\right] = \mathbb {E}_k [\mathbb {E}_{r,i}[\mathbbm {1}(F_k(\textsf{Sam}_i(r)), h(r,i))]]\\ =&\mathbb {E}_{r,i}[\mathbb {E}_k[\mathbbm {1}(F_k(\textsf{Sam}_i(r)), h(r,i))]] \le \max _{i} \mathbb {E}_{r}[\mathbb {E}_k[\mathbbm {1}(F_k(\textsf{Sam}_i(r)), h(r,i))]]\\ =&\max _i \frac{1}{2^\ell }\cdot \left( \sum _{r: \textsf{Sam}_i(r) = 0^\lambda } \mathbbm {1}(0^\lambda , h(r,i)) + \sum _{r: \textsf{Sam}_i(r) \ne 0^\lambda } \mathbb {E}_k[\mathbbm {1}(F_k(\textsf{Sam}_i(r)), h(r,i))]\right) \\ \le&\frac{T_M}{2^\ell } + \max _{r,i: \textsf{Sam}_i(r)\ne 0^\lambda } \mathbb {E}_k[\mathbbm {1}(F_k(\textsf{Sam}_i(r)), h(r,i))], \text { using } \mathbbm {1}(0^\lambda , h(r,i)) \le 1. \end{aligned}$$

#### Case BIPSW1

For any fixed *r*, *i* such that $\textsf{Sam}_i(r) \ne 0^\lambda$, the vector $k\cdot \textsf{Sam}_i(r)$ is uniformly distributed over $\{0,1\}^\lambda$, independently of *h*(*r*, *i*). As shown in [14], $1/3 - 1/2^\lambda \le \mathbb {E}_y[\mathbbm {1}(\textsf{map}(y), b)] \le 1/3 + 1/2^\lambda$ for any $b\in \{-1,0,1\}$, hence

$$\begin{aligned}&\mathbb {E}_k\left[ \Pr _{r,i}[F_k(\textsf{Sam}_i(r)) = h(r,i)]\right] \le \frac{T_M}{2^\ell } + \frac{1}{3} + \frac{1}{2^\lambda }. \end{aligned}$$

#### Case BIPSW2

For any fixed *r*, *i* such that $\textsf{Sam}_i(r) \ne 0^\lambda$, the value $\langle k, \textsf{Sam}_i(r)\rangle \bmod 6$ is uniform over $\mathbb {Z}_6$ (over the random choice of *k*) hence the bit $\lceil \langle k, \textsf{Sam}_i(r)\rangle \bmod 6 \rfloor _2$ is uniform over over $\{0,1\}$, independently of *h*(*r*, *i*). Therefore,

$$\begin{aligned}&\mathbb {E}_k\left[ \Pr _{r,i}[F_k(\textsf{Sam}_i(r)) = h(r,i)]\right] \le \frac{T_M}{2^\ell } + \frac{1}{2}. \end{aligned}$$

In the other direction:

$$\begin{aligned}&\mathbb {E}_k\left[ \Pr _{r,i}[F_k(\textsf{Sam}_i(r)) = h(r,i)]\right] \ge \min _{i} \mathbb {E}_{r}[\mathbb {E}_k[\mathbbm {1}(F_k(\textsf{Sam}_i(r)), h(r,i))]]\\ =&\min _i \frac{1}{2^\ell }\cdot \left( \sum _{r: \textsf{Sam}_i(r) = 0^\lambda } \mathbbm {1}(0^\lambda , h(r,i)) + \sum _{r: \textsf{Sam}_i(r) \ne 0^\lambda } \mathbb {E}_k[\mathbbm {1}(F_k(\textsf{Sam}_i(r)), h(r,i))]\right) \\ \ge&\frac{2^\ell - T_m}{2^\ell } \cdot \min _{r,i: \textsf{Sam}_i(r)\ne 0^\lambda } \mathbb {E}_k[\mathbbm {1}(F_k(\textsf{Sam}_i(r)), h(r,i))], \text { using } \mathbbm {1}(0^\lambda , h(r,i)) \ge 0. \end{aligned}$$

#### Case BIPSW1

Using again the fact that whenever $\textsf{Sam}_i(r) \ne 0^\lambda$, the vector $k\cdot \textsf{Sam}_i(r)$ is uniformly distributed over $\{0,1\}^\lambda$, since $\mathbb {E}_{y{\leftarrow }{{\$}}\{0,1\}^\lambda }[\mathbbm {1}(\textsf{map}(y), b)] \ge 1/3 - 1/2^\lambda$ for any *b*,

$$\begin{aligned}&\mathbb {E}_k\left[ \Pr _{r,i}[F_k(\textsf{Sam}_i(r)) = h(r,i)]\right] \ge \frac{2^\ell - T_m}{2^\ell } \cdot \left( \frac{1}{3} - \frac{1}{2^\lambda }\right) . \end{aligned}$$

#### Case BIPSW2

By a similar analysis as before,

$$\begin{aligned}&\mathbb {E}_k\left[ \Pr _{r,i}[F_k(\textsf{Sam}_i(r)) = h(r,i)]\right] \ge \frac{2^\ell - T_m}{2^{\ell +1}}. \end{aligned}$$

To finish the proof, as in [14], we use the Bienaymê-Chebyshev inequality (Lemma 2). In the following, we let $\delta$ be equal to $\frac{1}{3} + \frac{1}{2^\lambda }$ for BIPSW1 and to $\frac{1}{2}$ for BIPSW2. Bienaymê-Chebyshev yields

$$\begin{aligned} \Pr _k\left[ \Pr _{r,i}[F_k(\textsf{Sam}_i(r)) = h(r,i)] > \delta + \frac{T_M}{2^{\ell }} + \varepsilon \right] \le \frac{\sigma ^2}{\varepsilon ^2}, \end{aligned}$$

where $\sigma ^2$ denotes the variance of the random variable $X_k = \Pr _{r,i}[F_k(\textsf{Sam}_i(r)) = h(r,i)]$. Note that we can absorb $1/2^\lambda$ and $T_M/2^\ell$ in a term $\mathsf {negl(\lambda )}$. To conclude, it remains to bound the variance $\sigma ^2$. We first consider $\mathbb {E}_k[\mathbb {E}_{r,i}[\mathbbm {1}(F_k(\textsf{Sam}_i(r)), h(r,i))]^2]$, which is equal to

$$\begin{aligned}&\mathbb {E}_{K}[\mathbb {E}_{r,i}[\mathbbm {1}(F_k(\textsf{Sam}_i(r)), h(r,i))]\cdot \mathbb {E}_{r',i'}[\mathbbm {1}(F_k(\textsf{Sam}_{i'}(r')), h(r',i'))]]\\ \le&\max _{i,i'} \mathbb {E}_{r,r'}[\mathbb {E}_{K}[\mathbbm {1}(F_k(\textsf{Sam}_i(r)), h(r,i))]\cdot \mathbb {E}_{K}[\mathbbm {1}(F_k(\textsf{Sam}_{i'}(r')), h(r',i'))]]. \end{aligned}$$

Then, we decompose and bound the expectation over $r,r'$ below. Let $E_{i,i'}$ denote the set of pairs $(r,r')$ such that $r\ne r'$, $\textsf{Sam}_i(r) \ne 0^\lambda$, and $\textsf{Sam}_{i'}(r')\ne 0^\lambda$. We have for every $i,i'$

$$\begin{aligned}&\mathbb {E}_{r,r'}[\mathbb {E}_{k}[\mathbbm {1}(F_k(\textsf{Sam}_i(r)), h(r,i))]\cdot \mathbb {E}_{K}[\mathbbm {1}(F_k(\textsf{Sam}_{i'}(r')), h(r',i'))]]\\&\le \frac{1}{2^{2\ell }}\cdot \left( \sum _{ E_{i,i'}} \mathbb {E}_k[\mathbbm {1}(F_k(\textsf{Sam}_i(r)), h(r,i))]\cdot \mathbb {E}_k[\mathbbm {1}(F_k(\textsf{Sam}_{i'}(r')), h(r',i'))] + \sum _{ \bar{E}_{i,i'}} 1 \right) , \end{aligned}$$

where $\bar{E}_{i,i^{\prime }} := \{0,1\}^{2\ell }\setminus E_{i,i^{\prime }}$ and we sum over $(r,r^{\prime })\in E_{i,i^{\prime }}$ and $(r,r^{\prime })\in \bar{E}_{i,i^{\prime }}$, respectively. Then it holds that $|\bar{E}_{i,i^{\prime }}|\le$

$$ |\{(r,r^{\prime })\;:\; r \ne r^{\prime }\}| + |\{(r,r^{\prime })\;:\; \textsf{Sam}_i(r) = 0^{\lambda }\}|+|\{(r,r^{\prime })\;:\; \textsf{Sam}_{i^{\prime }}(r^{\prime }) = 0^{\lambda }\}| \le \tfrac{1+2T_M}{2^{\ell }}, $$

and for every $(r,r') \in E_{i,i'}$, since $r\ne r'$, $\textsf{Sam}_i(r) \ne 0^\lambda$, and $\textsf{Sam}_{i'}(r') \ne 0^\lambda$, we have

- **(for BIPSW1)** $k\cdot \textsf{Sam}_i(r)$ and $k\cdot \textsf{Sam}_{i'}(r')$ are uniformly and independently distributed over $\{0,1\}^\lambda$, therefore the expectation $\mathbb {E}_k[\mathbbm {1}(\textsf{map}(k\cdot \textsf{Sam}_i(r)), b) \cdot \mathbbm {1}(\textsf{map}(k\cdot \textsf{Sam}_{i'}(r')), b')]$ is bounded by $\delta ^2 = (1/3+1/2^{\lambda -1})^2$ for any $b,b'$.
- **(for BIPSW2)** $\lceil \langle k, \textsf{Sam}_i(r)\rangle \bmod 6 \rfloor _2$ and $\lceil \langle k, \textsf{Sam}_{i'}(r')\rangle \bmod 6 \rfloor _2$ are uniformly and independently distributed over $\{0,1\}$, , therefore the expectation $\mathbb {E}_k[\mathbbm {1}(F_k(\textsf{Sam}_i(r)), b) \cdot \mathbbm {1}(F_k(\textsf{Sam}_{i'}(r')), b')]$ is bounded by $\delta ^2 = (1/2)^2$ for any $b,b'$.

Combining the above observations, we get

$$\begin{aligned}&\mathbb {E}_{K}[\mathbb {E}_{r,i}[\mathbbm {1}(F_k(\textsf{Sam}_i(r)), h(r,i))]\cdot \mathbb {E}_{r',i'}[\mathbbm {1}(F_k(\textsf{Sam}_{i'}(r')), h(r',i'))]]\\ \le&\max _{i,i'} \mathbb {E}_{r,r'}[\mathbb {E}_{K}[\mathbbm {1}(F_k(\textsf{Sam}_i(r)), h(r,i))]\cdot \mathbb {E}_{K}[\mathbbm {1}(F_k(\textsf{Sam}_{i'}(r')), h(r',i'))]]\\ \le&\delta ^2 + \frac{1+2T_M}{2^\ell }. \end{aligned}$$

Eventually, using the definition of the variance,

$$\begin{aligned} \sigma ^2&\le \delta ^2 + \frac{1+2T_M}{2^\ell } - \left( \frac{2^\ell - T_m}{2^\ell } \cdot \delta \right) ^2\\&= \frac{1+2T_M}{2^\ell } + \delta ^2\cdot \frac{T_m}{2^\ell }\cdot \left( 2-\frac{T_m}{2^\ell }\right) \le \mathsf {negl(\lambda )}. \end{aligned}$$

We conclude the proof via a union bound over the *s* functions $h \in \mathcal {H}$. $\square$

### Security of VDLPN and EALPN against linear tests

#### Security against linear tests

Both the VDLPN and the EALPN assumptions are recent assumptions, introduced in [5] and [7], respectively. To provide support for VDLPN and EALPN, a natural approach is to analyze their security against standard attacks. In the context of LPN variants, the *linear test* framework (which has its roots in the seminal works of Naor and Naor [33] and of Mossel, Shpilka, and Trevisan [32], first explicitly put forth in [5] and further used in multiple subsequent works [6, 7, 19, 22]) provides a unified way to argue security against most standard attacks against LPN (such as Information-Set Decoding (ISD), or Blum-Kalai-Wassermann-style attacks [15], and many more). Concretely, an attack against LPN in the linear test framework proceeds in two stages:

1. First, a matrix *H* is sampled from the matrix distribution $\mathcal {H}$, and fed to the (unbounded) adversary $\textsf{Adv}$. The adversary returns a (nonzero) *test vector* $\textbf{v} = \textsf{Adv}(H)$.
2. Second, a noise vector $\textbf{e}$ is sampled from the noise distribution $\mathcal {N}$. The *advantage* of the adversary $\textsf{Adv}$ in the linear test game is the bias of the induced distribution $\textbf{v} \cdot H\cdot \textbf{e}^\intercal$.

We say that an instance of the syndrome decoding problem is *secure against linear test* if, with very high probability over the sampling of *H* in step 1, for any possible adversarial choice of $\textbf{v} = \textsf{Adv}(H)$, the bias of $\textbf{v} \cdot H\cdot \textbf{e}^\intercal$ induced by the random sampling of $\textbf{e}$ is negligible. Intuitively, the linear test framework captures any attack where the adversary is restricted to computing a linear function of the syndrome $\textbf{b}^\intercal = H\cdot \textbf{e}^\intercal$, but the choice of the linear function itself can depend arbitrarily on the code. Hence, the adversary is restricted in one dimension (it has to be linear in $\textbf{b}^\intercal$), but can run in unbounded time given *H*. Then, we say that an LPN-style assumption $(\varepsilon ,\delta )$*-fools* linear tests if

$$\begin{aligned} \Pr _H[\textsf{bias}(\mathcal {D}_H) > \delta ] \le \varepsilon , \end{aligned}$$

where $\mathcal {D}_H$ denotes the distribution which samples $\textbf{e}$ and outputs the LPN samples $H\cdot \textbf{e}$. The following shows that VDLPN cannot be broken by attacks from the linear test framework, which provides strong support for its security:

##### Theorem 16

([5], informal) $\textsf{VDLPN}(\lambda ,D,2^D)$ with $D= \Omega (\lambda )$ $(2^{-\Omega (\lambda )},2^{-\Omega (\lambda )})$-fools linear tests.

#### From security against linear tests to large minimum distance

A statement regarding security against linear tests is, under the hood, a statement about the minimum distance of a linear code whose parity-check matrix $H'$ is related to *H*. Below, we make this explicit for VDLPN and EALPN. In the case of VDLPN, it requires a little bit of work to exhibit the right matrix.

##### VDLPN

Given matrices $M_1, \cdots , M_n$ (for some *n*), we let $\textsf{BD}(M_1,\cdots , M_n)$ denote the block-diagonal matrix whose diagonal blocks are the $M_j$’s. Let $\mathcal {I}_i \in \mathbb {F}_2^{2^i \times 2^D}$ denote the horizontal concatenation of $2^{D-i}$ identity matrices of size $2^i\times 2^i$ (for any *i*), and let $B_i \leftarrow \textsf{BD}(\mathcal {I}_i, \cdots , \mathcal {I}_i)$ (where the number of blocks is equal to $\lambda$). We observe that the distribution $\mathcal {N}_\textsf{vd}(\textsf{par})$ can be equivalently described as follows: sample $\textbf{u}$ as the concatenation of $\lambda \cdot D$ length-$2^D$ unit vectors, and output $\textbf{e} = \textsf{BD}(B_1, \cdots , B_D) \cdot \textbf{u}$. Note also that $\textsf{BD}(B_1, \cdots , B_D)$ is a fixed matrix.

Now, sample $H \leftarrow \!\!\!{\$}\,\mathcal {H}_\textsf{vd}(\textsf{par})$ and define $H' \leftarrow H\cdot \textsf{BD}(B_1, \cdots , B_D)$. The VDLPN assumption is equivalent to the following assumption: given $(H', \textbf{b})$, it is hard to distinguish whether $\textbf{b}$ is random, or $\textbf{b} = H'\cdot \textbf{u}$, where $\textbf{u}$ is sampled as above. Then, we have the following simple lemma (proven in Lemma 17):

##### Lemma 17

The code generated by the rows of $H'$ has minimum distance at least $w = 2^D\ln (1/2.1\delta ) = \Omega (\lambda 2^D)$ with probability at least $1-\varepsilon$ over the choice of $H'$.

##### Proof

The proof is relatively simple. Assume towards contradiction that with probability larger than $\varepsilon$, the code generated by the rows of $H'$ does not have minimum distance *w*. This means that with probability $\varepsilon ' > \varepsilon$, there exists a vector $\textbf{v}$ such that $\textbf{v}^\intercal \cdot H'$ has Hamming weight less than *w*. Then,

$$\begin{aligned} \textsf{bias}_{\textbf{v}}(\mathcal {D}_{H}) = \left| 1/2 - \Pr _{\textbf{u}}[\textbf{v}^\intercal \cdot H'\cdot \textbf{u} = 1]\right| \ge \frac{1}{2}\cdot \left( 1-\frac{w}{\lambda D2^D}\right) ^{\lambda D} > \delta , \end{aligned}$$

which is a contradiction. Above, the bound on the bias follows from the piling-up lemma (Lemma 1) and the second inequality follows from the standard inequality $(1-1/n)^n \ge e^{-1}\cdot (1-1/n) > 0.99 e^{-1}$. $\square$

##### EALPN

In the case of EALPN, this is actually much more direct: the matrix $H'$ is simply equal to $H = M\cdot \Delta _N$ (where *M* is a random sparse matrix and $\Delta _N$ a lower triangle of ones). In fact, security against linear test is directly stated as a theorem about the minimum distance of the code spanned by *H* in [7]:

##### Lemma 18

([7], Theorem 3.10) Fix a parameter $c = \omega (\log N)$. The code generated by the rows of $H = M\cdot \Delta _N$ has minimum distance at least $\Omega (N)$, with probability at least $1-N^{-\omega (1)}$ over the choice of *H*.

#### A win-win result for PI-PRF security against linear tests

Equipped with the above results, we return to our initial question: how plausible is the assumption that the weak PCFs of [5, 19] and [7] are PI-PCFs? As it turns out, this question is equivalent to asking whether the wPRFs defined by VDLPN and EALPN are PI-PRFs. Since the main security argument supporting VDLPN and EALPN is that they are secure against linear tests, it is meaningful to ask whether the corresponding *pseudorandom-input* variants of VDLPN and EALPN resist linear tests, too.

By our above lemmas, this is equivalent to the following problem (we state it for VDLPN for concreteness, but the reasoning is similar for EALPN): given an admissible sampler $\textsf{Sam}$, if we sample each row $h_j$ of the matrix *H* as $\textsf{map}(x_j)^\intercal$, where $(x_1, \cdots , x_N) \leftarrow \!\!\!{\$}\,\textsf{Sam}$, does $H' = H\cdot \textsf{BD}(B_1, \cdots , B_D)$ have minimum distance $\Omega (\lambda )$? Let us denote $\mathcal {D}^r$ the distribution of $H'$ when random $x_1, \cdots x_N$ are used, and $\mathcal {D}^\textsf{pr}$ the distribution with $(x_1, \cdots , x_N) \leftarrow \!\!\!{\$}\,\textsf{Sam}$. Now, because $\textsf{Sam}$ is an admissible sampler, it holds that the distribution of $(x_1, \cdots , x_N)$ is computationally indistinguishable from random. Therefore, $\mathcal {D}^\textsf{pr}$ is computationally indistinguishable from $\mathcal {D}^r$, which samples codes with a minimum distance at least $\Omega (\lambda )$. That is, *no polynomial time adversary can distinguish* $H' \leftarrow \!\!\!{\$}\,\mathcal {D}^\textsf{pr}$ *from a code with a large minimum distance*. Using the terminology from [7, Definition 3.12], $\mathcal {D}^\textsf{pr}$ has a large *pseudodistance*.-

The existence of codes with a large gap between their pseudodistance and their actual minimum distance is an open problem which has received some attention in the literature. In particular, the hardness of finding a low-weight codeword, when it exists, is equivalent to the *binary SVP assumption* from [3]. The binary SVP assumption is known to have interesting consequences, such as the existence of collision-resistant hash functions with very low complexity (constant algebraic degree). Therefore, we obtain the following win-win result for PI-PCFs:

*Either the VDLPN-based candidate wPRF of* [5, 19] *is also a PI-PRF, or the binary SVP assumption holds with respect to the distribution* $\mathcal {D}^\textsf{pr}$*.* A similar win-win holds for the EALPN-based wPRF candidate of [7].

#### Key-agreement from VDLPN or EALPN

We further note that for the transformations to work, it suffices for the PI-PCF to be pseudorandom with respect to a *specific* admissible sampler $\textsf{Sam}$. Namely, let the sampler $\textsf{Sam}$ output $(x_1, \cdots , x_N) = \textsf{PRF}_K(z_1, \cdots , z_N)$, where $(z_1, \cdots , z_N)$ are (non-adaptively) defined by the sampler, and $\textsf{PRF}$ is a pseudorandom function (the key *K* of the PRF can be included in the PCF keys).

In Section 3.2, we show, analogously to Pietrzak-Sjödin [35], that if a wPRF is not also a $\textsf{PI}_{f}$-$\textsf{PRF}$, then there exists a key-agreement protocol. Now, let $\textsf{PRF}_K$ be a PRF which is pseudorandom under the VDLPN or EALPN assumption . Let us now instantiate the sampler $\textsf{Sam}$ with $\textsf{PRF}_K$ and assume that the wPRF is not also a $\textsf{PI}_{f}-\textsf{PRF}$. Then, under the VDLPN or EALPN assumption, the sampler $\textsf{Sam}$ instantiated with $\textsf{PRF}_K$ is an admissible sampler $\textsf{Sam}$, hence the construction from Section 3.2 yields a secure key-agreement protocol. Therefore, we get the following win-win result:

*Either the VDLPN-based candidate wPRF of* [5, 19] *is also a* $\text {PI}_{\textsf{PRF}_K}$*-PRF, or VDLPN implies key agreement. The same holds for EALPN.*

The problem of understanding whether VDLPN implies key agreement was explicitly put forth and studied in [6]. They showed that some natural approaches which use the Razborov-Smolensky lemma fail to yield key agreement, and could only obtain a positive result under an additional new assumption, called *random LPN is the hardest*.

## Data Availability

No datasets were generated or analysed during the current study.

## Footnotes

1 We here refer to a polynomial in $\lambda$ which is global for all $\textsf{elf}$ which are returned by $\textsf{ELF}.\textsf{Gen}(1^\lambda ,\cdot )$.

2 i.e. the adversary can only query the PRF non-adaptively

3 For PCFs, there is no need to store $k_\textsf{H}$ and $\textsf{elf}$ in a CRS: instead they can simply be sampled during PCF key generation, and added to each party’s key.

4 Recall that multiplication triples are linear shares [*a*], [*b*], [*c*] of some random multiplication triple $(a,b,c=ab)$ where $a,b{\leftarrow }{{\$}}\mathcal {R}$ where $\mathcal {R}$ is some ring. As shown by Beaver [9] parties holding linear shares of two different inputs $x,y\in \mathcal {R}$ can compute linear shares of $x\cdot y$ by: (1) locally computing shares of $\alpha =x-a$ and $\beta =y-b$ as $[\alpha ]\leftarrow [x]-[a]$ and $[\beta ]\leftarrow [y]-[b]$, (2) broadcasting the shares of $\alpha$ and $\beta$ to reconstruct these values, (3) locally setting $[x\cdot y]\leftarrow \alpha \cdot [y] + \beta \cdot [x] - \alpha \cdot \beta + [c]$.

5 Not to be confused with a *weak OPRF*, *a.k.a.* a *relaxed OPRF*, which is a relaxation of an OPRF introduced by Freedman et al. [24] which allows for some leakage of the key to the client.

6 Say a client and a server run two parallel instances of SFE of a wPRF, and the client queries a random input *x* in the first instance, and $x+1$ in the next: the inputs used by the client in each instance are random, but nevertheless correlated, and server security is not expected to hold.

7 A 1-out-of-2 bit-OT correlation can be defined as being sampled as a pair (of pairs) $(m_0, m_1)$ and $(\sigma , m_\sigma )$, where $(m_0, m_1)$ are the OT sender’s random messages in $\{0,1\}$, and $\sigma$ is the random choice bit given to the receiver.

8 More precisely, *correctness* (*i.e.* parties hold tuples of the form $(m_0, m_1)$ and $(\sigma , m_\sigma )$) is tied to the wPCF having *(weakly)* *OT**-correlated pseudorandom outputs*, while security (*i.e.* $m_{1-\sigma }$ is hidden from the receiver and $\sigma$ is hidden from the sender) is tied to *wPCF security*.

9 In fact, (interactive) OT extension is known to be in Minicrypt [29]. The minimal assumptions for silent OT extension are unknown.
